# Software for rapid time dependent ChIP-sequencing analysis (TDCA)

**DOI:** 10.1186/s12859-017-1936-x

**Published:** 2017-11-25

**Authors:** Mike Myschyshyn, Marco Farren-Dai, Tien-Jui Chuang, David Vocadlo

**Affiliations:** 1 Department of Molecular Biology and Biochemistry, 8888 University Drive, Burnaby, BC V5A 1S6 Canada; 2 Chemistry, Simon Fraser University, 8888 University Drive, Burnaby, BC V5A 1S6 Canada

**Keywords:** ChIP-seq, Time course experiment, Bioinformatics, Protein-DNA binding kinetics, Data modeling, Curve fitting, Statistical analysis, Genomic feature correlations

## Abstract

**Background:**

Chromatin immunoprecipitation followed by DNA sequencing (ChIP-seq) and associated methods are widely used to define the genome wide distribution of chromatin associated proteins, post-translational epigenetic marks, and modifications found on DNA bases. An area of emerging interest is to study time dependent changes in the distribution of such proteins and marks by using serial ChIP-seq experiments performed in a time resolved manner. Despite such time resolved studies becoming increasingly common, software to facilitate analysis of such data in a robust automated manner is limited.

**Results:**

We have designed software called Time-Dependent ChIP-Sequencing Analyser (TDCA), which is the first program to automate analysis of time-dependent ChIP-seq data by fitting to sigmoidal curves. We provide users with guidance for experimental design of TDCA for modeling of time course (TC) ChIP-seq data using two simulated data sets. Furthermore, we demonstrate that this fitting strategy is widely applicable by showing that automated analysis of three previously published TC data sets accurately recapitulates key findings reported in these studies. Using each of these data sets, we highlight how biologically relevant findings can be readily obtained by exploiting TDCA to yield intuitive parameters that describe behavior at either a single locus or sets of loci. TDCA enables customizable analysis of user input aligned DNA sequencing data, coupled with graphical outputs in the form of publication-ready figures that describe behavior at either individual loci or sets of loci sharing common traits defined by the user. TDCA accepts sequencing data as standard binary alignment map (BAM) files and loci of interest in browser extensible data (BED) file format.

**Conclusions:**

TDCA accurately models the number of sequencing reads, or coverage, at loci from TC ChIP-seq studies or conceptually related TC sequencing experiments. TC experiments are reduced to intuitive parametric values that facilitate biologically relevant data analysis, and the uncovering of variations in the time-dependent behavior of chromatin. TDCA automates the analysis of TC ChIP-seq experiments, permitting researchers to easily obtain raw and modeled data for specific loci or groups of loci with similar behavior while also enhancing consistency of data analysis of TC data within the genomics field.

**Electronic supplementary material:**

The online version of this article (10.1186/s12859-017-1936-x) contains supplementary material, which is available to authorized users.

## Background

In recent years ChIP-seq has become a hallmark strategy to define genomic loci that are bound by particular proteins [1–4]. Genome organization and regulation of gene expression are dynamic processes and enable adaptation to changes in cellular signaling, physiology, and environmental cues, therefore, there has been increasing interest in understanding the time-dependent changes in binding of proteins to the genome. Such studies depend on quantifying the number of sequencing reads at a given locus as a function of time in a series of parallel experiments. Using such data, changes in the number of sequencing reads at specific loci can be compared to changes at other loci, allowing one to evaluate changes in the abundance of proteins associated with specific genomic loci. Accordingly, such analyses are of increasing interest because uncovering genomic loci that are particularly responsive or impervious to a diverse range of stimuli will enable improved understanding of mechanistic basis behind the dynamic changes within the genome that enable adaptive responses.

Several reports have described TC ChIP-seq and ChIP-seq-like studies performed using a variety of techniques. The current scope of TC experiments has involved metabolic feeding of unnatural amino acids [5], induction of engineered genes bearing epitope tags [6–13], stimulus with known effectors of protein-DNA binding [14, 15], induction of DNA cleavage by activation of proteins fused to nucleases [16, 17], investigating nucleosome position changes using the assay for transposase-accessible chromatin followed by sequencing (ATAC-seq) procedure [18, 19], and examining the repair of DNA damage [20]. The development of novel tools to enable TC ChIP-seq analysis of new targets is an area of growing interest and such methods will facilitate a host of studies that should uncover new mechanisms contributing to the activation and repression of genes.

Although new TC ChIP-seq experimental strategies continue to be developed [21], the strategies for analysis of TC data vary widely. Indeed, there is no standard method for analysis within the field and this stems in part from the lack of software dedicated to such analyses. To our knowledge, there are three publications that offer analysis scripts for TC ChIP-seq data processing, mostly with limited functionality, documentation, applicability and none of these offer modeling options [9, 14, 16]. Manual analysis strategies are more common. Researchers have estimated rates of turnover at genomic loci by manually fitting sequencing coverage data at each locus over time to an inverse of a negative exponential formula [5]. Strategies to calculate sequencing coverage at loci in TC ChIP-seq experiments over time using a multi-linear regression has also been explored [10, 13]. Other TC ChIP-seq analysis strategies instead focused simply on trends in the coverage of sequencing reads over time at loci of interest [16, 20]. Strategies involving data fitting are appealing because they enable researchers to reduce large amounts of complex data to a limited set of theoretically important values. Furthermore, using data fitting methods ensures that data at all loci are fit in a consistent manner, increasing the consistency of analyses and avoiding experimenter bias. However, complicating issues can arise when data cannot be fit by the proposed functions or if the model is overly simple. These problems can lead to loss of important information and missing insights that could otherwise be gleaned. Given the decreasing costs of sequencing, coupled with the high value of TC data for understanding physiological responses manifesting within the genome, TC studies are an area of growing interest. Accordingly, simple automated methods that facilitate analysis of such data will facilitate the adoption of TC methods by researchers new to the TC field as well as by non-specialists considering implementing ChIP-seq studies in their own research programs.

Here we describe the development and validation of software that greatly facilitates analysis of a wide range of TC data in a robust automated manner. We call this software the Time-Dependent ChIP-Sequencing Analyser (TDCA). TDCA analyzes the sequencing read coverage at a series of time points and uses this data to calculate protein binding half-lives at genomic loci by modeling TC sequencing coverage to sigmoidal curves. We provide a comprehensive manual containing full algorithm details as well as installation procedures with our software, which is publicly available at: www.github.com/TimeDependentChipSeqAnalyser/TDCA. The following manuscript focuses on describing the accuracy, versatility, and utility of TDCA. We demonstrate the accuracy and versatility of TDCA by testing simulated data sets, as well as by replicating key findings and providing new insights from previously published data sets that were obtained using diverse methods. These data sets include: 1) TC ChIP-seq of doxycycline inducible HA-tagged histone 3.3 (H3.3) variant in MEF cells [10], 2) Chromatin endogenous cleavage followed by sequencing (ChEC-seq) of Abf1 in yeast [16], and 3) eXcision repair sequencing (XR-seq) on (6–4)pyrimidine-pyrimidone photoproducts ([6–4]PP) in a normal fibroblast cell line (NHF1) and a DNA damage prone cell line (CS-B) in humans [20]. Data analysis by TDCA yields intuitive parameters that describe behavior at genomic loci and offers customizable analysis with publication-ready graphical outputs, thus making TDCA of particular value for researchers.

## Implementation

### Strategy

Given that the amount of any specific protein bound to any given genomic locus must have an upper limit to its occupancy, we felt that using an inverse of a negative exponential function for data modeling should accurately reflect the eventual saturation or steady-state occupancy that should occur at loci over time. We also reasoned that protein binding to genomic regions should reach a lower limit defined by either complete vacancy or, in some cases, a low basal level. Finally, we reasoned that many methods applied to TC ChIP-seq, including for example the induction of tagged proteins, will involve a delay in responses that are not accounted for by a simple inverse negative exponential function. To account for this induction period, while incorporating the upper and lower limits of protein binding to the genome, we opted to fit data to sigmoidal curves. Fitting to a sigmoidal curve readily enables the definition of parameters that also define the speed at which occupancy of a given protein changes at any genomic locus. Finally, we also considered that such sigmoidal curves may be asymmetric since, for example, in systems where induction of expression of a protein of interest is used to control the extent of protein binding, then the extent of recruitment to a locus may be initially limited by protein abundance but then rapidly accelerate as protein production is induced. This type of system would result in loss of rotational symmetry between the curve before and after the inflection point. From a biological perspective, this asymmetry reflects that the rate at which protein binding occurs at the locus and varies as being unequal on either side of the inflection point. This inequality could arise from a positive/negative feedback response to the protein expression/binding process or may be caused by changes in the experimental conditions - for instance if researchers wished to see the effect of protein binding rates in response to some given stimulus partway through a TC experiment. To account for such scenarios, we introduced the option of introducing an asymmetry parameter to describe such behavior. We also expect that as sequencing becomes less expensive TC studies will become commonplace and more time points will be acquired to allow more precise modeling. We therefore considered that such sigmoidal fits should yield basic parameters that define the properties of binding of a given protein of interest at any genomic locus. These biologically relevant parametric outputs are reported to users as raw data. This approach accordingly enables users to reduce complex sequencing experiments to a few key features, clarifying research questions and enabling focused data analysis.

### Core algorithm

TDCA models [22] normalized sequencing coverage [23] to four parameter (4P) or five parameter (5P) sigmoidal curves, at user specified loci, across multiple ChIP-seq TC experiments. TDCA accepts TC sequencing data in BAM file format and loci coordinates in standard BED file format. Raw sequencing data can be aligned to a reference genome using a variety of published software [24, 25] and converted to BAM files using SAMtools [23]. Loci at which precipitated proteins bind DNA at significant levels, or ChIP-seq “peaks”, can be defined using published software [26, 27] or through custom analysis strategies. The equation and description of parameters for 4P and 5P sigmoids are shown in (Eq. 1).

1 $$ y=d+\frac{a-d}{{\left(1+{e}^{b\left(x-c\right)}\right)}^f} $$

Where,

*a* = Lower asymptote (baseline protein binding).

*b* = Incorporation rate index (IRI, a measure of the slope at the inflection point).

*c* = Inflection point when *f* = 1 (also the time at which the curve reaches the TTI when *f* = 1).

*d* = Upper asymptote (maximal protein binding).

*f* = Asymmetry factor (A measure of the rotational symmetry about the inflection point. For 0 < *f* < 1: the y-value for the inflection point occurs closer to the lower asymptote. For *f* = 1: the rate of increase is the same as the rate of decrease such that the inflection point occurs exactly in between the lower and upper asymptote (the curve is symmetric). For *f* > 1: y-value for inflection point occurs closer to the upper asymptote).

The inflection point of Eq. 1 is the point on the sigmoidal curve at which a change in the direction of curvature occurs. Mathematically, this is given by the root of the second derivative of Eq. 1, given by Eq. 2:

2 $$ x=-\left(\frac{\mathit{\ln}(f)- bc}{b}\right) $$

Equation 2 defines the value of x at which the root of the second derivative (and consequently the inflection point) occurs. When *f* = 1 the root occurs at *x* = *c*. Inputting this equivalency into Eq. 1 yields a y-value of (*a* + *d*)/2, the mean value between upper and lower asymptote, which we use to define the turnover time index (TTI). However, for any other value of *f* the inflection point is shifted away from c and is dependent on both the values of *b* and *f*. For cases in which *f* does not equal 1, the TTI value in such asymmetric cases is obtained directly by solving for the value of *x* for which *y* = (*a* + *d*)/2. Note that the recommended and default setting for *f* is fixed to 1 and changing this setting should only be done by users with a clear biological rationale since a variable *f* may not be relevant. For a graphical representation of the effect of the *f* value on curve behavior, as well as interpretation of these effects, refer to “Core Algorithm Description” section of the manual.

During fitting, each locus in a TC ChIP-seq experiment is defined as one of six characteristic TDCA categories of change in sequencing coverage as a function of time. These six categories of behavior are defined as follows:

1. Rises: Sequencing coverage increases over time and data are modeled to a single 4P sigmoid having a negative incorporation rate index.
2. Falls: Sequencing coverage decreases over time and data are modeled to a single 4P sigmoid with a positive incorporation rate index.
3. Hills: Sequencing coverage increases and then decreases over time and data are modeled to two 4P sigmoids - a rise then a fall.
4. Valleys: Sequencing coverage decreases and then increases over time and data are modeled to two 4P sigmoids - a fall then a rise.
5. Undefined: Loci that do not display the behavior of the previous categories but are nevertheless modeled as either a single rise or fall.
6. Eliminated: Loci that are predicted to behave as a certain category but do not.

We have enabled TDCA to normalize sequencing coverage data before modeling. This normalization can be done in two ways. Firstly, the coverage values at each locus are normalized by the maximum sequencing coverage at non-peak loci for all time points collected in a TC series. Using non-peak loci enables capturing levels of true background sequencing. Additionally, TDCA can accommodate use of an input standard for normalization of data sets obtained at each time point. ‘Input’ refers to sequencing data for a control experiment wherein the protein-DNA complexes are not immunoprecipitated by a specific antibody and the sequencing results therefore provide a baseline sequencing coverage distribution. If input control data is provided, the input is normalized in the same manner except the sequencing coverage across the entire genome is used since there are no expected peaks. Sequencing coverage at each time within the input data is then subtracted from experiment data to a lower limit of zero. However, applying this subtraction strategy can lead to zero inflation depending on the quality of the input files that are used. To combat this potential problem, we have enabled TDCA to analyze pre-normalized read counts, which allows users to apply the most appropriate normalization strategy to their particular experiment [28–30]. In the TDCA manual, we provide an example of how users can achieve normalized read counts using DiffBind [31], which incorporates the popular programs DESeq2 [32] and edgeR [33], which account for overdispersion. To assist users who wish to limit the weight put on observations with large counts, which can lead to greater variances, we have also incorporated an option to model user TC sequencing data using a Poisson model instead of by least-squares fitting. TDCA has the capacity to handle any number of replicate data sets as well as any amount of input data. Notably, in order to accommodate novel spike-in normalization strategies that are emerging [34, 35], we also provide users with the option to normalize data to a defined set of values (see manual for details). Overall, the normalization strategies implemented here were designed to keep TDCA compatible for analysis of a broad variety of TC sequencing data, even as novel normalization strategies are developed.

**Fig. 1:**
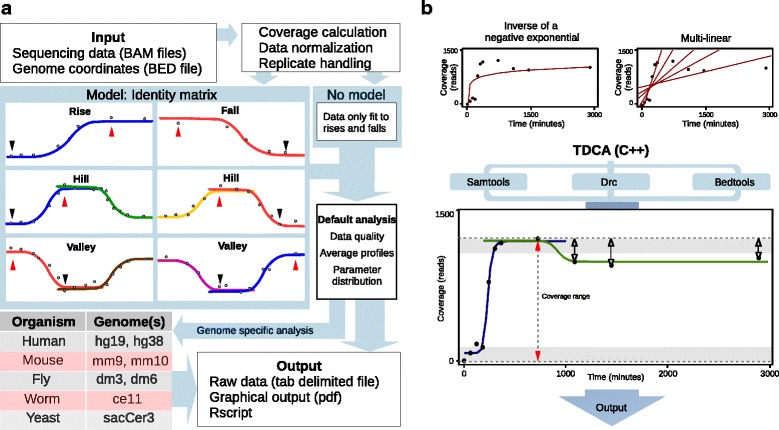
TDCA analysis work flow, requirements, and performance. **a** Simplified work flow. Required input data are genomic coordinates in BED format and folders containing BAM TC sequence files. TDCA normalizes data based on total sequencing coverage of each time point and also handles input files and replicates using additional normalization procedures. Loci can be modeled as the following categories of signal change: rise, fall, hill, or valley. An identity matrix that predicts loci category is based on the time at which absolute minimum sequencing coverage (black arrows) and absolute maximum sequencing coverage (red arrows) occurs as set by user defined thresholds. Each sigmoid color indicates a rise or fall with different combinations of absolute maximum and absolute minimum coverage positions in time with genuine leading and trailing points. Alternatively, users can model all their data to a single sigmoidal curve. The resulting parameters from data fitting are then reported to the user along with raw sequencing coverage calculations. Graphical output is provided to the user which can be enriched by specifying genome and genes. R scripts are provided in case users would like to change the look of default figures. **b** Plots show sequencing coverage (y-axis) over time (x-axis) at loci for coordinates of chromosome 1:5,012,338–5,013,264 obtained from a H3.3 ChIP-seq experiment [10] using previously applied modeling strategies of inverse negative exponential (upper left) and multi-linear (upper right), and the sigmoidal fitting used by TDCA (lower). TDCA requires on terminal access to SAMtools [23] for sequencing coverage calculation of BAM files, BEDTools [37] for BED file manipulations, and R with the drc [22] package for curve fitting. In the example shown here, parameters that govern data modeling by TDCA can be fine-tuned to result in either a single or double sigmoid. The lower and upper horizontal dashed lines represent absolute minimum coverage and absolute maximum coverage values, respectively. The overall sequencing coverage range at a locus is shown as a vertical dashed line with red arrows. In this case, the three data points marked with white arrows exceed the plateau range threshold (gray boxes) and are defined as genuine absolute maximum trailing data points. This results in double sigmoid modeling as shown here. Parameters for both sigmoids are reported to users. The plateau range threshold and leading/trailing threshold could be adjusted such that the locus is modeled to a single sigmoid

To model data, we have designed TDCA to use a prediction algorithm that is based on the times at which the normalized absolute minimum and maximum sequencing coverage values are observed at each locus. TDCA checks if there are either trailing data points (occurring later in time) and leading data points (occurring earlier in time) for the time points containing the absolute minimum and maximum sequencing coverage to identify lower and upper asymptote boundaries and to determine if the behavior at a locus is a candidate for modeling using a double sigmoid as seen in “hills” or “valleys” or whether the behavior at the locus is described by a “rise” or “fall” and modeled by a single sigmoid, Fig. 1 (a). We enabled TDCA to use a user-defined “plateau range threshold” and “leading/trailing points threshold”, which control the tolerated variation in sequencing coverage that can be used to define a lower or upper asymptote boundary. Briefly, the plateau range threshold allows users to define the tolerated differences in sequencing coverage that is used to determine if the leading and trailing data points are within range to be considered asymptotes (i.e. if the differences are simply fluctuations of data points which have reached a plateau), or if the points are in fact changing meaningfully over time. If the latter is the case, then these data points are defined as genuine leading or trailing time point that permits defining an upper or lower asymptote boundary (for each side of the valley or hill) and corresponding assignment of behavior at a locus to either a hill or valley. The user defined leading/trailing points threshold allows users to define how many genuine leading or trailing data points (as determined by the plateau range threshold) are necessary to shift the modeling of a loci from a single to a double sigmoid, Fig. 1 (b). The ability of TDCA to model a single locus to a range of specific categories based on a user-adjustable prediction algorithm allows one to gain important insights from available data. Furthermore, the categories we have defined are biologically relevant, as shown through description provided below for the TDCA automated analyses of several published data sets. For additional clarification, the TDCA manual contains a more detailed description of the leading/trailing points threshold and the plateau range threshold.

After the categorization of each locus is completed, TDCA models the data at each locus and the time points used are separated according to the category of behavior predicted. If the modeling result does not match the prediction, the locus is eliminated. For example, if a locus is predicted to model as a rise but is in fact modeled as a fall, the locus is eliminated from downstream analysis. This procedure provides a two-fold verification of locus behavior that effectively eliminates loci that are false positives. A visual representation of our algorithm is shown in Fig. 1 (a) and the dependencies for operation of TDCA as well as a visual of the TDCA modeling process in Fig. 1 (b). We have also optimized TDCA to operate using parallel processor libraries (Additional file 1: Figure S1).

TDCA can also model time course sequencing data using linear regression. This may be useful in situations where constant rates of binding of a protein or other measurable factor is observed over time at a given locus. Constant coverage over time would result in the locus being modeled to a line with a relatively flat slope and low overall residuals. This output can be directly compared with the residuals from the sigmoidal fits to enable users to evaluate suitability of the modeling. Graphical outputs of these measurements are all provided by TDCA to facilitate analysis of TC sequencing data.

TDCA provides the results of the modeling as an output file. Standard errors of each parameter of the modeled curves are also provided. These standard errors provide measures of the accuracy with regard to the parameters that are estimated. Accordingly, these errors can be used to gauge the reliability of the modeled parameters. In particular, confidence intervals can be calculated using the standard errors, which can give users a deeper understanding of the accuracy of the estimated values offered for the various parameters. These errors should also be used to guide iterative experiments that lead to their reduction and also replicate findings in entirely independent sets of experiments. Standard errors obtained using different modeling functions can also be compared to assess the most appropriate model for the experimental design. We have created TDCA to offer various graphical outputs [36], predominantly using the turnover time index (TTI), which is the inflection point obtained from the modeled data adjusted by the asymmetry factor, or simply the inflection point in the case of the default 4P curve fitting. The TTI is indicative of the binding half-life of a protein at a particular locus and, for this reason, we find it to be a biologically interesting variable on which to focus attention.

## Results and discussion

### Analysis of simulated ChIP-seq time course data

To test the accuracy of TDCA, we generated simulated TC ChIP-seq data describing both rises and falls (see Methods for details). Briefly, we varied different parameters for 1000 loci located on three chromosomes of the *Drosophila* genome. On chromosome 2 L we assigned loci to vary in the time of the inflection point, defined as the turnover time index (TTI) and the magnitude of the slope at the TTI, defined as the incorporation rate index (IRI). On chromosome 2R we varied the length of the peaks. On chromosome 3R, we varied the position of the upper asymptote, which defines the coverage of sequencing at loci. Calculated values for each of the 3000 loci were converted into sequencing coverage values for 11 different time points [37], and different random noise was added to each time point using standard methods [38]. We provide tracks of the simulated data [39] (Additional file 1: Figure S2 (a-c)), which are summarized in Additional file 1: Figure S3 (a-d). Our simulated data generation method allowed us to generate a constant level of noise which we believed would reflect the random background noise observed within real experiments (Additional file 1: Figure S4 and S5). It is important to note that the application of this random noise, however, does not account for the extent of biological variation at loci which is generally greater than random noise and depends very much on the experimental system. Although this simulated noise may not reflect the noise distribution in specific biological experiments, we envisioned that these simulated data sets would be useful in allowing assessment of the accuracy in modeling parameters in the absence of biological variability at loci and help stimulate users to think about the design of experiments in terms of parameters such as, most importantly, the frequency of data collection. We analyzed the simulated data using TDCA and focused on how well it could model the position of the TTI, since this is a biologically interesting parameter equivalent to the time at which half of protein binding change at a particular locus occurs. To perform this study, we evaluated the percent difference of the true inflection point based on the simulated calculations with the TTI calculated by TDCA using the TC data augmented with noise.

**Fig. 2:**
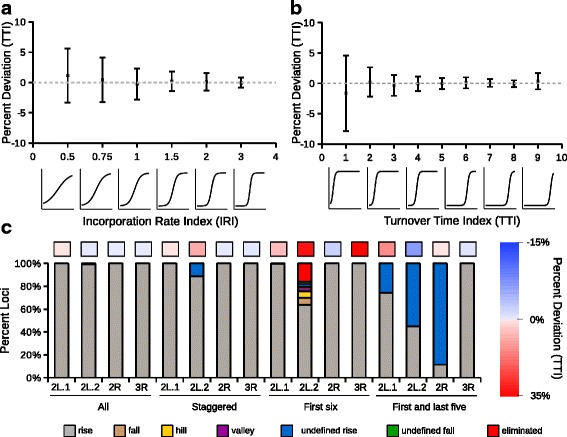
Simulated data analysis. **a** Percent deviation of TDCA modeled TTI to true TTI of simulated rises on chromosome 2 L.1 using all time points binned by the absolute IRI. Representations of true data for different IRI values is shown underneath the deviation plots. **b** Percent deviation of TDCA modeled TTI to true TTI of simulated rises on chromosome 2 L.2 using all time points binned by the true TTI value. Representations of true data for different TTI values is shown underneath the deviation plots. **c** Identification of loci categories in simulated rise data using different combinations of time points (all points, staggered points, first six points, and first and last five points). Upper boxes indicate average percent deviation of TDCA modeled TTI to true TTI with a scale shown to the right

Analysis of the 3000 loci with simulated rise and fall data revealed that the TTI modeled by TDCA accurately predicts the true inflection point of the large majority of data (Additional file 1: Figure S6). TDCA shows increased percent deviation from the true inflection point when data behaves more linearly, with a low absolute incorporation rate index (Additional file 1: Figure S6 (a) and (b)), or when inflection points occur very near the first or last time points for which data is obtained (Additional file 1: Figure S6 (c) and (d)). This behavior is summarized for simulated data describing rises on the first part of chromosome 2 L (2 L.1), where the incorporation rate index systematically changes across loci (Fig. 2 (a)) and on the second part of chromosome 2 L (2 L.2), where inflection points systematically change across loci (Fig. 2 (b)). Interestingly, we also observed more accurate TTI predictions of chromosome 2R loci with higher relative saturation (Additional file 1: Figure S6 (e) and (f)). We reasoned that this behavior arises from the added noise contributing less significantly to data with overall greater sequencing coverage, since greater sequencing coverage would improve the signal to noise ratio. Therefore, both noise and sequencing coverage are important factors to consider in TDCA modeling accuracy. Finally, we found that peak length had no noticeable effect on accuracy of modeling (Additional file 1: Figure S6 (g) and (h)). Based on these analyses, we note that there are important factors to consider in TDCA modeling accuracy, and indeed analysis of TC ChIP-seq data in general, including the extent of noise, sequencing coverage, and the time points collected in the context of expected changes in protein binding to the genome. Regardless, deviation of fitted models to the simulated data sets revealed small (±10%) differences and we therefore consider the overall modeling accuracy of TDCA to be satisfactory.

Given the value of having adequate time points to flank the TTI as noted above, we next evaluated how accurately TDCA would model our simulated data sets when only select time points were used. This analysis should provide useful guidance as to how many and at which times one should collect experimental data to realize reliable modeling of data by TDCA. We tested evenly staggered time points (0, 2, 4, 6, 8, and 10), the first six time points (0, 1, 2, 3, 4, and 5), and the first single and last five time points (0, 6, 7, 8, 9, and 10). These tests stem from practical situations that may arise at specific loci, where a researcher may have collected fewer time points (staggered), may have unknowingly ended collection prematurely (first six), or may have missed a block of time points or preferred to collected later data sets (first and last five).

Using these sparser simulated data sets, we analyzed the percent deviation of the true simulated inflection point to the TTI modeled by TDCA at each locus (Additional file 1: Figure S7, S8 and S9). We found that the percent deviation was most significant at loci that contained true inflection points that were beyond the last available time point or within gaps of available time points. For example, using staggered time points we noticed that loci on chromosome 2 L.2 with inflection points at time point 1 increased in percent deviation (Additional file 1: Figure S7 (c) and (d)). When we modeled data using the first six time points, there was an expected and clear loss in accuracy for loci at chromosome 2 L.2 having a TTI at a time greater than time point 5, which was the last time point included in this truncated analysis (Additional file 1: Figure S8 (c) and (d)). Similarly, we noticed during analyses of the data sets containing data for the first time point along with the data for the last five time points, a notable loss in accurate modeling of the TTI at loci having inflection points that occurred within the gap of time points (1–5) (Additional file 1: Figure S9 (c) and (d)). Interestingly, when analyzing the truncated data set containing only the first six time points, there was a larger deviation in accurate modeling of TTI for loci in chromosomes 2R and 3R, with inflection points of 4.5 and 5.5, respectively, in simulated rise data compared to simulated fall data (Additional file 1: Figure S8 (e-h)). We reasoned that this effect stemmed from difficulty TDCA had in pinpointing the upper asymptote of rises, whereas those of falls could more easily be determined due to the constraint of requiring placement of the lower asymptote at a non-negative value.

Given that current recommendations regarding TC sequencing experiments calls for late time points to satisfy saturation of captured loci [40], modeling late TTI values should not be a major problem for researchers so long as this recommendation is followed. In order to circumvent issues in modeling early TTI values, we recommend limited preliminary studies that enable selection of suitable time points chosen to flank the TTI and then perform deeper sequencing studies for TC ChIP-seq experiments and modeling.

In our simulated TC experiments, we also describe the accuracy of predictions returned by TDCA with regard to locus categorization for each simulated data set (Additional file 1: Figure S10). Fundamentally, these results reflect the accuracy of the prediction of inflection points. As shown (Fig. 2 (c)), the locus category prediction for simulated rises is most sporadic at chromosome 2 L.2 when using only the first six time points. TDCA has difficulty predicting loci category when using only the first single time point along with the last five time points. This situation leads to a large occurrence of loci assigned as being undefined, however, the correct category of signal change is predicted (rises and falls are categorized as undefined rises and falls, respectively). Overall, loci that are correctly predicted by TDCA as being in their true category are more likely to be accurately modeled, indicating an important aspect of category predictions that should help guide favorable experimental TC ChIP-seq study design.

### Analysis of inducible HA-tagged histone H3.3 variant in MEF cells

**Fig. 3:**
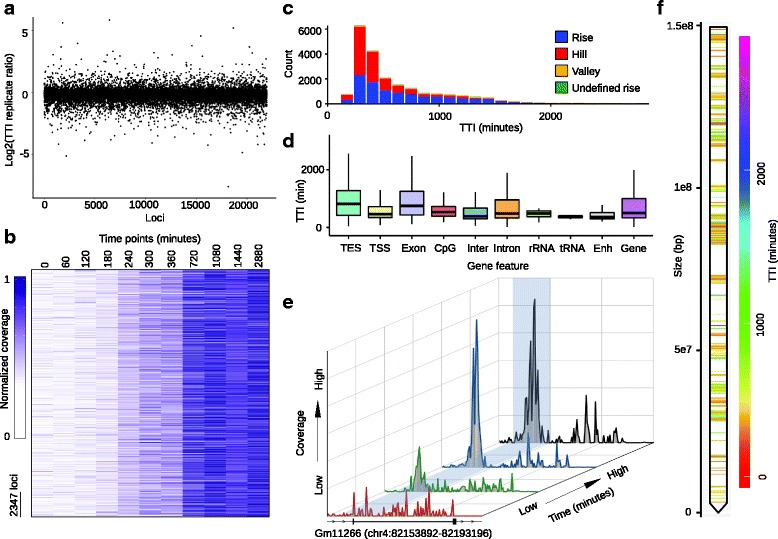
TDCA analysis of data from reported HA-tagged H3.3 doxycycline inducible TC ChIP-seq experiments performed in MEF cells. **a** Log2 ratio of TTI values from replicate 1 and 2 across each locus. **b** Coverage heat map across time points for 23,475 loci. Data for each locus are normalized from 0 (absolute minimum coverage) to 1 (absolute maximum coverage) so that loci can be compared with each other by visual inspection. **c** Distribution of loci that display signal increase are grouped within the defined modeling categories. TTI is shown on the x-axis and loci count on the y-axis. **d** Distribution of TTI values for loci that display increased signal at specific genome features. Lower lines, lower part of box, midline, upper part of box, and upper line are 1st quartile, 2nd quartile, median, 3rd quartile and 4th quartile respectively. The following genomic features are displayed: 3’UTR to 1000 bp downstream (TES), 5’UTR to 1000 bp upstream (TSS), coding exons (Exon), CpG islands (CpG), intergenic regions (Inter), introns (Intron), rRNA genes (rRNA), tRNA genes (tRNA), enhancers (Enh), and whole genes (Gene). **e** 3D plot of sequencing coverage for the gene Gm1266 (chr4:82,153,892–82,193,196). Black boxes indicate exons, dark lines indicate introns, and lines with arrows indicate 1000 bp upstream and downstream regions. Highlighted region shows the position of two loci with TTI values of 338.9 and 322.3 min. **f** Ideogram heat map of chromosome 6. Bands indicate the positions of H3.3 bound loci and the color scale indicates the TTI values rises and inclines of hills

To showcase key features of our program we analyzed a robust TC ChIP-seq experiment performed using an engineered MEF cell line that produces HA-tagged H3.3 variant in the presence of doxycycline in a time dependent manner [10]. This data set contains two independent replicates at each of 11 time points, as well as an input control. We analyzed the replicates separately and found that the log2 TTI ratio of replicates across loci predominantly centered around zero (Fig. 3 (a)) with 73.4% of loci within ±20% and 94.4% of loci within ±50% of the reported TTI value (Additional file 1: Figure S11). This analysis supports good reproducibility of the replicate experiments.

We next proceeded to analyze H3.3 loci using both replicates, along with the input control. Included in the default graphical output of TDCA is a genome wide heat map of normalized sequencing coverage across time points (Fig. 3 (b)). This is a useful chart to visualize the overall quality of data. We observed a general trend of increasing sequencing coverage over time (Fig. 3 (b)), which is expected as doxycycline treatment leads to a gradual increase of the tagged H3.3 and its recruitment to the genome. Other default graphs generated by TDCA includes a pie chart showing the percentage of loci that are assigned into one of the six TDCA categories of behavior and a bar chart showing the percent incidence of absolute minimum and absolute maximum sequencing coverage values over all collected time points (Additional file 1: Figure S12). We found that the H3.3 TC data contained 49.7% rises and 41.2% hills, accounting for 90.9% of loci. Importantly, the occurrence of decreasing signal after a maximum (defined as a being a hill) was also observed in the original analysis of the data [10], supporting the accuracy of the automated analysis and locus categorization performed by TDCA. We also observed an increased occurrence of absolute minimum coverage near the early time points and an increased occurrence of absolute maximum coverage at late time points. Overall, the quality charts support the expectation of increased signal over time.

TDCA offers many default graphs to facilitate data analysis and interpretation. Of particular use is a count of loci that fall within binned TTI regions, which can be separated by the category assigned for a given locus (Fig. 3 (c)). During analysis of this H3.3 data set, we observed a right tailed skewed distribution of TTI values centered around 300 min. From this observation, we noticed that the distribution of the TTI of the incline of the hills were faster than those of rises. This is an interesting and previously unobserved property of these data that may have functional significance that merits closer study. TDCA also automatically displays average profiles for each category of locus and we illustrate this output showing the relevant categories, hills and rises, for this H3.3 data set (Additional file 1: Figure S13).

We expanded the customizable built in mouse gene feature library within TDCA to include analysis of loci comprising genes that encode tRNA and rRNA [41], as well as loci encompassing enhancers [42] (see manual). These gene features were previously analyzed and found to exhibit unusually fast turnover of H3.3. Here, using TDCA, we rapidly replicated these results in a single automated step and include the distribution of TTI at other default gene features included in TDCA at loci that show an increase in signal change (Fig. 3 (d)).

TDCA also provides the useful option of graphing, in a compressed 3D format, the normalized read coverage at specific loci. Figure 3 (e) shows the 3D profile of the gene Gm11266 (chr4:82,153,892–82,193,196), which contains two loci bound by H3.3, which according to the raw data output, have TTI values of 338.9 and 322.3 min. As shown, saturation is observed at the last two compressed sequencing coverage values. Conversely, the 3D profile of the gene Sgk1 (Additional file 1: Figure S14), which also contains two loci bound by H3.3, does not appear to become saturated with tagged H3.3. Consultation with the raw data supports this conclusion, revealing TTI values of 1868.4 and 1732.5 min for Sgk1. Overall, these 3D profiles are visually informative and provide users with a quick and intuitive way to examine the behavior of genes of particular interest.

Lastly, TDCA provides the distribution of loci to which H3.3 is bound along chromosomes, along with their TTI as an additional dimension shown in color as illustrated here for chromosome 6 (Fig. 3 (f)) and genome wide (Additional file 1: Figure S15 (a)). This ideogram heat map allows users to quickly scan the genome-wide distribution of their loci while simultaneously considering TTI values to decide if clustering analyses, such as the discovery of hotspots describing clusters of fast (low TTI) or slow (high TTI) loci exist within the data set. We binned the mouse genome into 200,000 bp bins and overlapped H3.3 loci at each bin. We found 30 bins that contained 30 or more H3.3 loci, which we defined as being clusters. We then plotted the average TTI and corresponding standard deviation within each of these clusters (Additional file 1: Figure S15 (b)). Not surprisingly, since H3.3 shows a relatively bland TTI distribution, we find no drastic differences in TTI averages at clusters after considering the standard deviation. However, some clusters contain much smaller standard deviations than others, which suggests that some clusters are more tightly co-regulated in terms of H3.3 binding or turnover.

### Analysis of Abf1 time course ChEC-seq in yeast

Recently, an interesting ChIP-seq-like technique called ChEC-seq, escaping the general requirement of using antibodies for IP and for DNA fragmentation, has been described. This strategy relies on genetically engineered proteins of choice fused to calcium dependent endonucleases. Researchers can study the kinetics of binding of these fusion proteins along the genome by treating cells with calcium at various time points and for varying times. Although not a ChIP-seq experiment *per se*, the resulting data is completely amenable for analysis by TDCA.

We decided to test the performance of TDCA on a published ChEC-seq experiment in which an Abf1 fusion protein was used in yeast [16]. This data set contains progressively longer treatments with calcium. This experiment should theoretically result in gradually increasing levels of DNA fragments that in time reach some upper limit, which would result in the TCDA loci categorization of rises to predominate. However, the authors did note that for some loci, there was an increase in signal over time and then a disappearance, theoretically resulting in the TCDA loci category of hills. Because TDCA can model loci in the same data set as different categories a clear advantage can be gained using this software for automated analysis. We analyzed the Abf1 ChEC-seq data set and found that 11,715/12351 loci (94.9%) identified as rises or hills which contained positive TTI values on the signal increase modeled sigmoid. This encouraged us to proceed to reproduce key findings in the published data set to prove the accuracy of TDCA, as well as to highlight novel insights gained only through TDCA usage.

Previously, the Abf1 data set was categorized into two major clusters by k-means clustering and these categories were defined as being fast and slow. This categorization was based on whether the time point at which the absolute maximum coverage after normalization occurred either early (fast category) or late (slow category). Focus was then directed on analyzing DNA sequence motifs and their abundance at both fast and slow loci. The authors found that fast and slow loci showed a tendency to contain high and low scoring motifs, respectively. Notably, TDCA uncovered a more complex distribution of the kinetic binding patterns of Abf1, as shown in the distribution of TTI values (Additional file 1: Figure S16 (a)). When we used k-means clustering [43] to bin the TTI values obtained using TDCA into fast and slow categories we replicated the key observation that there is an increase in the motif scores of fast loci compared to slow. This effect, however, was more modest and not as great as previously reported based on the time of absolute maximum sequencing coverage (Additional file 1: Figure S16 (b)). Notably, we also found that the previously clustered fast and slow loci do show an overall lower and higher TTI distribution, respectively (Additional file 1: Figure S16 (c)). TDCA is therefore in general agreement with this previous analysis strategy and the reported Abf1 data set.

**Fig. 4:**
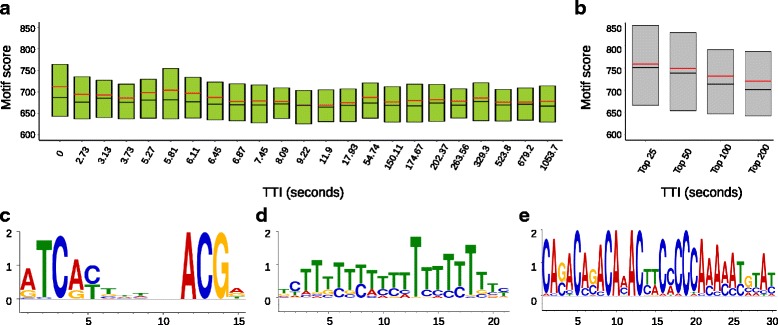
TDCA aided analysis of ChEC-seq studies of Abf1 binding in yeast reveals predominantly hills and rises. **a** Distribution of motif scores of Abf1 ChEC-seq loci that are ordered from fastest to slowest based on their TTI values and ranked into bins containing groups of 500 loci. The lower boundary for each bin is defined by the lowest TTI value in seconds and is shown on the x-axis. Black midline indicates median and red midline indicates average. Quartiles 2 and 3 are lower and upper fractions of the box divided by the median. **b** Distribution of motif scores of Abf1 ChEC-seq loci ordered by TTI of the top 25, 50, 100, and 200 fastest TTI loci. Black midline indicates median and red midline indicates average. Quartiles 2 and 3 are lower and upper fractions of the box divided by the median. Top scoring motifs (most significant) of the first (**c**), second (**d**), and third (**e**), bins of 1000 Abf1 ChEC-seq loci ordered by their TTI values

We next took the clustering based on the time point at which the absolute maximum coverage after normalization occurred to its greatest limit by creating the smallest possible clusters. These smallest clusters are simply each time point used. We observed a general trend of increasing motif averages as the bins neared zero (Additional file 1: Figure S16 (d)). Binning loci based on the TDCA obtained TTI value corresponding to the time points of calcium treatment did not show as great a trend for average motif scores as previously described (Additional file 1: Figure S16 (e)). We reasoned that this apparent difference was due to a large proportion of loci containing TTI values occurring within 1 min (Additional file 1: Figure S16 (a)). We therefore ordered loci based on fastest to slowest TTI values and created bins containing 1000 loci. The average motif scores at these ordered bins re-captured similar average motif scores of clustered data based on the time point at which the absolute maximum coverage occurred (Additional file 1: Figure S16 (f)). Strikingly, when we decreased the bin size to 500 loci (Fig. 4 (a)), we observed an even greater average motif score at the fastest TTI bin, with local minima and maxima bin clusters. This resolution could not be obtained using the previously published strategy. We show that there are progressively dramatic leaps in the average motif scores as we observe the top 200, 100, 50, and 25 TTI loci. This marked increase in the motif score that stems from narrowing the bin size of the loci having the greatest TTI values highlights the importance of increasing resolution and speaks to the utility and accuracy of the TTI value in analyzing data sets.

Lastly, we ordered all loci based on their TTI from fastest to slowest and created bins of 1000 loci for which we then produced motifs (Additional file 1: Figure S17). We were able to reproduce specific motifs [44] at loci having early TTI (Fig. 4 (c)), which eventually reduced to poly-A repeats, as noted in the initial report [16]. Because of our increased resolution, we also captured additional motifs that were not previously observed (Fig. 4 (d-e)). Interested researchers would easily be able to pursue this type of discovery using the high level of automation and customizability offered by TDCA.

### Analysis of time course XR-seq on [6–4]PP in NHF1 and CS-B human cells

In humans, UV damaged DNA is removed through the action of the nucleotide excision repair pathway [45]. By monitoring DNA repair following UV treatment in a TC XR-seq experiment it has been shown that the time at which excision occurs after UV exposure varies depending on the locus and that excised fragments, which can be identified and quantified by sequencing, degrade over time [20]. This observation suggests that resulting TC sequencing data analyzed by TDCA should categorize predominantly as either rises and hills, depending on the rate of degradation of excised DNA fragments. We used macs [26] to determine loci containing excised [6–4]PP, using the longest time point (240 min) and the shortest time point (5 min) as the signal and baseline, respectively. We viewed this process as leading to the identification of loci that release excision products at a relatively late time. Accordingly, we found that 96.2% (7565/7860) of NHF1 and 97.2% (5121/5268) CS-B loci are identified as rises.

To showcase the plateau range threshold option of TDCA we described previously, we performed an analysis of [6–4]PP loci using a range of plateau range thresholds. As expected, we found there to be a modest but consistent increase in the number of loci that were categorized as rises as the plateau range threshold became looser (Additional file 1: Figure S18 (a)), for both NHF1 and CS-B cell lines. We also used TDCA for analysis with input files containing sets of loci that had been called by macs using different *p*-value thresholds [26]. While holding the plateau range threshold at a constant value and specifying more stringent macs *p*-values, there was a general increase in the percent of loci that identified as rises (Additional file 1: Figure S18 (b)). This is meaningful since the loci called at lower p-values should be more accurate.

In order to show that peaks called using the longest time point (240 min) and the shortest time point (5 min) as the signal and baseline, respectively, specifically result in rises, we analyzed three different randomly permuted [37] coordinates of [6–4]PP loci in NHF1 and CS-B cells while keeping the coverage normalization constant (see methods). We found that the identity of these random loci were not specifically enriched in rises (Additional file 1: Figure S18 (c)). This type of analysis is important to help demonstrate the specificity of behavior at loci having user defined coordinates.

To demonstrate the behavior of all [6–4]PP loci we analyzed the top 1% 500 bp bins in NHF1 and CS-B that showed the greatest change in sequencing coverage over time for chromosomes 21 and 22, as done previously [20]. We found that 48.8% NHF1 and 50.6% CS-B loci identified as rises, suggesting that excision products from DNA resulting from excision at some loci may persist for longer than others, which are presumably degraded more quickly by nucleases. Notably, we also found that 24.7% NHF1 and 28.0% CS-B loci identified as falls, although this is likely an artifact since the first time point at which sequencing was performed was only 5 min after UV exposure. Alternatively, however, falls may represent exceptionally quickly excised loci and may be of functional significance.

**Fig. 5:**
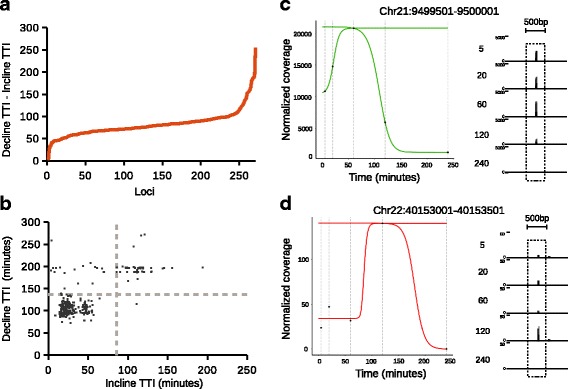
TC XR-seq analysis in NHF1 cells. **a** 271 hills from chromosomes 21 and 22 ordered by the difference of their TTI_fall_ and TTI_rise_. This difference (TTI_fall_ - TTI_rise_) reflects the clearance rate of DNA excised from these loci. **b** Scatter plot of the signal increase (incline) TTI (TTI_rise_) and signal decrease (decline) TTI (TTI_fall_) of 271 hills from chromosomes 21 and 22. Two clusters are apparent from this analysis that indicating either slow (top right quadrant) or fast (bottom left quadrant) excision of DNA at loci. **c** TDCA modeling of the locus at chromosome 21:9,499,501–9,500,001. Individual coverage values are shown as black dots and can be visually seen in the sequencing tracks shown to the right. **d** As in (**c**) except for the locus at chromosome 22:40,153,001–40,153,501

To showcase TDCA, we decided to focus our analysis on the hills present in the NHF1 cell line. 13.6% (271/1990) of loci from this data set were categorized by TDCA as hills, which is a much greater fraction than that found in CS-B cells (41/1990 = 2.5%). Plotting the difference in the TTI values for the declines and inclines of each hill in NHF1 cells revealed an average difference and standard deviation of 83.2 ± 19.9 min (Fig. 5 (a)). We find this is a reasonably tight time range and we hypothesize that the clearance of excision products at loci that identified as hills occurred within a certain limited time frame.

Next, we wanted to determine if the TTI values of the hill inclines and hill declines were correlated in some manner. At each locus, the inclines and declines seemed to cluster by visual inspection, which was corroborated by k means clustering [43] (Fig. 5 (b)). We plotted a hill that had a relatively fast incline (TTI_rise_) and decline (TTI_fall_) as defined by the TTI values for each fitted sigmoid (Fig. 5 (c)). We also plotted a hill with a relatively slow incline (TTI_rise_) and decline (TTI_fall_) as defined by the TTI values for each fitted sigmoid (Fig. 5 (d)). These loci share similar clearance rates of excised product, as defined by the difference of fall and rise TTI of hills (TTI_fall_-TTI_rise_) yet excision appears to start at different times. This observation could potentially direct researchers to identify the molecular basis for why loci within this data set cluster in this way and why some loci seem to show delayed excision. Notably, TDCA greatly enabled these analyses in an automated and intuitive manner and we anticipate TDCA can similarly be applied to facilitate analysis of a wide variety of experimental TC sequencing data studies.

### Discussion

We describe a novel algorithm that we have developed called TDCA, which models changes in sequencing coverage of individual loci within time course (TC) ChIP-seq, or conceptually related experiments, as a function of time. The behaviors of these changes are categorized as rises, falls, hills, and valleys. Such sigmoidal modeling of TC ChIP-seq data has, to our knowledge, not been performed. We believe such modeling of TC ChIP-seq data has a reasonable basis in underlying biological principles and that the outputs obtained from TDCA provide intuitively relevant parameters. Our analysis of three published and publicly available data sets support this view, illustrating that rises and hills are biologically meaningful ways to describe behavior of TC ChIP-seq data. Although the data sets explored here differ widely in terms of the experimental means used to obtain them, the outputs provided by TDCA proved in all cases to be readily applicable. Indeed, our automated analyses of these data sets reveal the speed by which TDCA can be applied to obtain insights that are of potential biological importance. Using TDCA, we rapidly recapitulated key findings from these data sets as well as being able to detect previously unobserved behaviors of potential biological significance. Notably, other published data sets also support falls as a biologically meaningful behavior [13] and we anticipate that valleys may be observed in the future as the scope of such TC sequencing studies grow and new experimental designs are applied.

TDCA offers many customizable options, such as the ability to tune modeling parameters, include genome specific analyses, and specify normalization constants. These properties confer on TDCA considerable versatility and should permit its use in analysis of nearly any type of TC ChIP-seq study or conceptually related TC sequencing experiments. TDCA is also amenable for analysis of developmental ChIP-seq studies. Considering the available well-documented protocols [46] and the global changes found in developmental ChIP-seq studies [47], this should be a straightforward application for TDCA, and provide useful insights into such experiments. Furthermore, we recognize that investigators could readily use TDCA to model dose-response ChIP-seq experiments. Treating cells with inhibitors of chromatin associated proteins for different time periods or in a dose-dependent manner followed by ChIP-seq of the inhibited proteins or their chromatin marks could, for example, distinguish between inhibitor sensitive and insensitive loci. Furthermore, novel advances in mapping genome associated small molecules [48] applied in dose-response ChIP-seq experiments could also complement or provide new insights into dose-responsive behavior of genomic loci. TDCA could be applied to both time- and dose-responsive ChIP-seq strategies with only minor adjustments to the labeling of the x-axis.

TDCA does have some limitations with regard to the type of behavior expected in a time-course experiment. Any kind of behavior that does not fall into a typical fall, rise, hill or valley model category may not be accurately fitted. For instance, in a TC experiment where multiple hills or valleys occur within TC data for one locus, TDCA would not be able to properly model such oscillating data. Another scenario that would present problems would be a rise that reaches a plateau that is followed by another rise; this step-like behavior would be modeled as a single rise. The analogous step fall-plateau-fall behavior would also be poorly modeled. Though these scenarios are currently unknown and seem improbably, users will ultimately need to consider if their experimental design is suitable to analysis by TDCA.

To guide users in experimental design and to highlight the strength and limitations of TDCA, we have also analyzed simulated data. These analyses provide guidance for the number of time resolved data sets needed for successful analysis. They also help define which distributions of time resolved data points are beneficial for proper and reliable modeling. Accordingly, the results of these efforts, along with previously recommended protocols [40] should be considered when designing TC ChIP-seq experiments or other conceptually related TC studies.

The default analysis produced by TDCA and custom analysis using the TTI output described here are intended to stimulate activity in the field of TC ChIP-seq by providing computation support for analysis of resulting TC data. This work is also intended to provide conceptual impetus to more deeply consider analysis strategies and enable rapid exploration of various analysis parameters so as to enable the community to glean as many insights as are available within the growing stream of data being generated within this important and rapidly developing field.

## Conclusions

To stimulate further research in the area of time course (TC) ChIP-seq experiments, we have developed the first robust automated tool for analysis of such data. TDCA accepts sequence alignment data in BAM file format and loci in BED format. The graphical and raw data output provided by TDCA provides users with biologically relevant data and will facilitate research as well as inspire future effort in this growing area of study. While we have described the use of TDCA in the context of ChIP-seq experiments to monitor protein binding to the genome, the term protein is used throughout for simplicity and analysis by TDCA is applicable to analysis of any molecular feature associated with the genome that can be detected in a selective manner. Moreover, we show that TDCA works to quickly capture the key findings of published datasets and has the potential to be applied to many TC sequencing experiments. Accordingly, as strategies applicable to TC sequencing studies develop, existing strategies improve, and costs of sequencing continue to decline, we expect TDCA will prove broadly useful for a wide range of new experiments as well as providing a benchmark system to help guide optimization of data collection.

## Methods

### TDCA design and dependencies

SAMtools [23] is required for coverage calculation of BAM files. The samtools depth -r command is used for this and is called to the terminal within TDCA. The bedtools intersect [37] command is used for the genome specific analysis. User defined peak coordinates are intersected with genome feature BED files and the TTI values are reported as a boxplot. TDCA uses the R package dose-response curve (drc) [22] for data modeling to a sigmoidal curve. The generation of graphs requires the following R packages: ggplot2 [36], scales, and grid. In addition, plot3D and rgl are required for the construction of the 3D scatterplot of user specified genes when the -3d flag is called. Much of TDCA is parallelized including commands called by TDCA such as BEDTools, SAMtools and drc. We used openmp for this which requires an appropriate compiler (see www.openmp.org).

### Simulated data generation

We assigned 1000 loci to three chromosomes in the *Drosophila* genome, 2 L, 2R, and 3R. The inflection points of loci on the first half of chromosome 2 L (2 L.1) were fixed at 5 with variable incorporation rate indices (IRIs) of: −0.5, −0.75, −1.0, −1.5, −2.0, and −3.0 for rises and the corresponding absolute values for falls. The inflection points of loci on the second half of chromosome 2 L (2 L.2) were set to: 1, 2, 3, 4, 5, 6, 7, 8, and 9, with incorporation rate indices set to −3 for rises and the corresponding absolute value for falls. The inflection point of loci on chromosome 2R and 3R were held constant at 4.5 and 5.5, respectively, with incorporation rate indices set to −1.5 for rises and the corresponding absolute value for falls.

With these calculated values in mind we created BAM files that satisfied the required coverage for 11 time points (0–10, relative units). To do this, we iteratively concatenated coordinates of loci to each other, for each time point, and converted to a BAM file (bedtools bedToBam, [37]). Each concatenation increased coverage by the length of the bed file coordinate, therefore, the concatenation never truly reached the exact value of required coverage. This was intentional and permitted an intrinsic aspect of noise. However, the intrinsic noise was found to be negligible, so different simulated background noise was merged with each time point using simulated 1X coverage of the entire *Drosophila* genome using ART [38].

We ran TDCA on simulated rise and fall data using all time points (0–10), staggered time points (0, 2, 4, 6, 8, and 10), the first six time points (0–5), and the first and last five time points (0 and 6–10). This was done using the command: tdca -bed 3000-loci.bed -bam < folder_name>. We converted BAM files to bedGraph (BDG) files (bedtools genomecov, [37]), added header files, and used UCSC to visualize tracks.

### Analysis of external data

The general procedure followed to obtain processable external data was to convert SRA files into fastq format using the fastq-dump command from the SRA toolkit [49]. The files were then aligned to the appropriate reference genome using the bwa mem command from the Burrows-Wheeler aligner [24]. The resulting sequence alignment map files were then converted to binary, sorted, cleared of duplicate reads, and indexed using the samtools view -bS, sort, rmdup, and index commands, respectively [23]. Sources of data retrieval and additional specific processing instructions are listed below. Default TDCA parameters were used (tdca -bed loci.bed -bam < folder_name > −L5) unless otherwise specified.

### Inducible HA tagged H3.3

H3.3 replicate 1 TC data was obtained from GEO accession numbers GSM1246648-GSM1246659. H3.3 replicate 2 TC data was obtained from GEO accession numbers GSM1246660-GSM1246670. Input TC data was obtained from GEO accession numbers GSM1246671-GSM1246682. Data was aligned to the mm9 genome. Time points at 72 h for rep1 and input were used for peak calling only, not in tdca analysis. Peaks were called using macs2 callpeak q-value of 1e-7 [26]. For the expansion of the TDCA genome feature library, tRNA and rRNA gene coordinates were curated from UCSC [41] and enhancer loci from VISTA Enhancer [42].

### Abf1 ChEC-seq

Free MNase ChEC-seq (input) data was obtained from GEO accession numbers GSM1647289-GSM1647299. Abf1 ChEC-seq data was obtained from GEO accession numbers GSM1647300-GSM1647312. Data was aligned to the sacCer3 genome. Peaks were not called for the ChEC-seq data. Instead, loci were obtained from supplementary data set 1 from the original manuscript [16]. TDCA was run using -t 0 because hills emerged as early as the second time point (resulting in one genuine absolute maximum trailing data point in some cases). K-means clustering was performed using mlpack_kmeans [43]. We used motif scores from the original manuscript so that our results would be highly comparable. For motif discovery, we used MEME-ChIP [44].

### [6–4]PP XR-seq

Replicates 1 and 2 in NHF1 cells (6–4)PP XR-seq were obtained from GSM1985857-GSM1985866. Replicates 1 and 2 in CS-B cells (6–4)PP XR-seq were obtained from GSM1985867-GSM1985874. Data was aligned to the hg19 genome.

Slow loci were called with macs2 using cells that had 240 min to heal after UV treatment as the experiment file and cells that had 5 min to heal after UV treatment as the control file. Peaks were called for replicates separately, then concatenated and merged to remove redundancy. For the plateau range threshold analysis and the shuffled loci analysis, peaks called with a *p*-value threshold of 1e-3 were used. For the p-value analysis, peaks were called using *p*-values of, 1e-3, 1e-4, 1e-4, and 1e-5. TDCA analysis with shuffled loci was performed using the -dm flag and a file containing the same normalization coverage values as non-shuffled loci so that the shuffled and non-shuffled loci could be directly compared.

For loci that displayed variable signal across time, we binned the hg19 genome into 500 bp bins and analyzed the top 1% loci at chromosomes 21 and 22 that had the largest range in sequencing coverage at each bin after normalizing for variability in total sequencing depth.

## Availability and requirements

Time Dependent ChIP-Sequencing Analysis (TDCA) is freely available at: www.github.com/TimeDependentChipSeqAnalyser/TDCA under the GNU General Public License v3.0. TDCA is written in c++ and R and was extensively tested on Linux operating systems. TDCA requires terminal access to UNIX commands and installed dependencies such as samtools, bedtools, and various R packages including drc.

## Additional file

Additional file 1: SI for Software for Rapid Time Dependent ChIP-Sequencing Analysis (TDCA). (PDF 6149 kb)

## Abbreviations

[6–4]PP: (6–4)pyrimidine-pyrimidone photoproducts
4P: Four parameter in reference to a sigmoid curve
5P: Five parameter in reference to a sigmoid curve
BAM: binary alignment map
BED: browser extensible data
ChEC-seq: Chromatin endogenous cleavage followed by sequencing
ChIP-Seq: Chromatin immunoprecipitation followed by sequencing
IRI: Incorporation rate index
TC: Time course
TDCA: Time-Dependent ChIP-Sequencing Analyser
TTI: Turnover time index
XR-seq: eXcision repair sequencing

## References

[1] Johnson DS, Mortazavi A, Myers RM, Wold B (2007). Genome-wide mapping of in vivo protein-DNA interactions. Science.

[2] Barski A, Cuddapah S, Cui K, Roh TY, Schones DE, Wang Z (2007). High-resolution profiling of Histone Methylations in the human genome. Cell.

[3] Robertson G, Hirst M, Bainbridge M, Bilenky M, Zhao Y, Zeng T (2007). Genome-wide profiles of STAT1 DNA association using chromatin immunoprecipitation and massively parallel sequencing. Nat Methods.

[4] Mikkelsen TS, Ku M, Jaffe DB, Issac B, Lieberman E, Giannoukos G (2007). Genome-wide maps of chromatin state in pluripotent and lineage-committed cells. Nature.

[5] Deal RB, Henikoff JG, Henikoff S. Genome-wide kinetics of nucleosome turnover determined by metabolic labeling of histones. Science. 2010;328:1161–4. Available from. http://www.ncbi.nlm.nih.gov/pubmed/20508129. 10.1126/science.1186777 PMC2879085 20508129

[6] Mito Y, Henikoff JG, Henikoff S. Histone replacement marks the boundaries of cis-regulatory domains. Science. 2007;315:1408–11. Available from: http://www.ncbi.nlm.nih.gov/pubmed/17347439. 10.1126/science.1134004 17347439

[7] Mito Y, Henikoff JG, Henikoff S (2005). Genome-scale profiling of histone H3.3 replacement patterns. Nat. Genet.

[8] Dion MF, Kaplan T, Kim M, Buratowski S, Friedman N, Rando OJ (2007). Dynamics of replication-independent histone turnover in budding yeast. Science.

[9] Lickwar CR, Mueller F, Hanlon SE, McNally JG, Lieb JD (2012). Genome-wide protein-DNA binding dynamics suggest a molecular clutch for transcription factor function. Nature.

[10] Kraushaar DC, Jin W, Maunakea A, Abraham B, Ha M, Zhao K (2013). Genome-wide incorporation dynamics reveal distinct categories of turnover for the histone variant H3.3. Genome Biol.

[11] Ha M, Kraushaar DC, Zhao K (2014). Genome-wide analysis of H3.3 dissociation reveals high nucleosome turnover at distal regulatory regions of embryonic stem cells. Epigenetics Chromatin.

[12] Yildirim O, Hung JH, Cedeno RJ, Weng Z, Lengner CJ, Rando OJ (2014). A system for genome-wide Histone variant dynamics in ES cells reveals dynamic MacroH2A2 replacement at promoters. PLoS Genet.

[13] Deaton AM, Gómez-Rodríguez M, Mieczkowski J, Tolstorukov MY, Kundu S, Sadreyev RI (2016). Enhancer regions show high histone H3.3 turnover that changes during differentiation. elife.

[14] wa Maina C, Honkela A, Matarese F, Grote K, Stunnenberg HG, Reid G (2014). Inference of RNA polymerase II transcription dynamics from chromatin Immunoprecipitation time course data. PLoS Comput Biol.

[15] Fiorito E, Sharma Y, Gilfillan S, Wang S, Singh SK, Satheesh SV (2016). CTCF modulates estrogen receptor function through specific chromatin and nuclear matrix interactions. Nucleic Acids Res.

[16] Zentner GE, Kasinathan S, Xin B, Rohs R, Henikoff S (2015). ChEC-seq kinetics discriminates transcription factor binding sites by DNA sequence and shape in vivo. Nat Commun.

[17] Grünberg S, Henikoff S, Hahn S, Zentner GE. Mediator binding to UASs is broadly uncoupled from transcription and cooperative with TFIID recruitment to promoters. EMBO J 2016;35:2435–2446. Available from: http://emboj.embopress.org/lookup/doi/10.15252/embj.201695020%0A, http://www.ncbi.nlm.nih.gov/pubmed/27797823. 10.15252/embj.201695020 PMC5109241 27797823

[18] Mueller B, Mieczkowski J, Kundu S, Wang P, Sadreyev R, Tolstorukov MY (2017). Widespread changes in nucleosome accessibility without changes in nucleosome occupancy during a rapid transcriptional induction. Genes Dev.

[19] Schep AN, Buenrostro JD, Denny SK, Schwartz K, Sherlock G, Greenleaf WJ (2015). Structured nucleosome fingerprints enable high-resolution mapping of chromatin architecture within regulatory regions. Genome Res.

[20] Adar S, Hu J, Lieb JD, Sancar A. Genome-wide kinetics of DNA excision repair in relation to chromatin state and mutagenesisProc Natl Acad Sci U S A. 2016;201603388. Available from: http://www.pnas.org/lookup/doi/10.1073/pnas.1603388113, http://www.ncbi.nlm.nih.gov/pubmed/27036006, http://www.pubmedcentral.nih.gov/articlerender.fcgi?artid=PMC4839430. 10.1073/pnas.1603388113 PMC4839430 27036006

[21] Liu T-W, Myschyshyn M, Sinclair DA, Cecioni S, Beja K, Honda BM (2016). Genome-wide chemical mapping of O-GlcNAcylated proteins in Drosophila Melanogaster. Nat Chem Biol.

[22] Ritz C, Baty F, Streibig JC, Gerhard D (2015). Dose-response analysis using R. PLoS One.

[23] Li H, Handsaker B, Wysoker A, Fennell T, Ruan J, Homer N (2009). The sequence alignment/map format and SAMtools. Bioinformatics.

[24] Li H, Durbin R (2009). Fast and accurate short read alignment with burrows-wheeler transform. Bioinformatics.

[25] Zaharia M, Bolosky W, Curtis K. Faster and more accurate sequence alignment with SNAP. arXiv Prepr. 2011;1–10. Available from: http://arxiv.org/abs/1111.5572.

[26] Zhang Y, Liu T, Meyer CA, Eeckhoute J, Johnson DS, Bernstein BE (2008). Model-based analysis of ChIP-Seq (MACS). Genome Biol.

[27] Heinz S, Benner C, Spann N, Bertolino E, Lin YC, Laslo P, et al. Simple combinations of lineage-determining transcription factors prime cis-regulatory elements required for macrophage and B cell identities. Mol Cell. 2010;38:576–589. Available from: doi:10.1016/j.molcel.2010.05.004. 10.1016/j.molcel.2010.05.004 PMC2898526 20513432

[28] Liang K, Keleş S (2012). Normalization of ChIP-seq data with control. BMC Bioinformatics.

[29] Diaz A, Park K, Lim DA, Song JS (2012). Normalization, bias correction, and peak calling for ChIP-seq. Stat Appl Genet Mol Biol.

[30] Lun ATL, Smyth GK (2016). Csaw: a bioconductor package for differential binding analysis of ChIP-seq data using sliding windows. Nucleic Acids Res.

[31] Ross-Innes CS, Stark R, Teschendorff AE, Holmes KA, Ali HR, Dunning MJ (2012). Differential oestrogen receptor binding is associated with clinical outcome in breast cancer. Nature.

[32] Love MI, Huber W, Anders S (2014). Moderated estimation of fold change and dispersion for RNA-seq data with DESeq2. Genome Biol.

[33] Robinson MD, McCarthy DJ, Smyth GK. edgeR: a bioconductor package for differential expression analysis of digital gene expression data. Bioinformatics 2010;26:139–140. Available from: http://www.ncbi.nlm.nih.gov/pubmed/19910308, http://www.pubmedcentral.nih.gov/articlerender.fcgi?artid=PMC2796818. 10.1093/bioinformatics/btp616 PMC2796818 19910308

[34] Bonhoure N, Bounova G, Bernasconi D, Praz V, Lammers F, Canella D (2014). Quantifying ChIP-seq data: a spiking method providing an internal reference for sample-to-sample normalization. Genome Res.

[35] Egan B, Yuan C-C, Craske ML, Labhart P, Guler GD, Arnott D (2016). An alternative approach to ChIP-Seq normalization enables detection of genome-wide changes in Histone H3 lysine 27 Trimethylation upon EZH2 inhibition. PLoS One.

[36] Wickham H. ggplot2. Elegant graph. Data Anal. 2009; Available from: http://link.springer.com/10.1007/978-0-387-98141-3.

[37] Quinlan AR, Hall IM (2010). BEDTools: a flexible suite of utilities for comparing genomic features. Bioinformatics.

[38] Huang W, Li L, Myers JR, Marth GT (2012). ART: a next-generation sequencing read simulator. Bioinformatics.

[39] Kent WJ, Sugnet CW, Furey TS, Roskin KM, Pringle TH, Zahler AM (2002). The human genome browser at UCSC. Genome Res.

[40] Lickwar CR, Mueller F, Lieb JD (2013). Genome-wide measurement of protein-DNA binding dynamics using competition ChIP. Nat Protoc.

[41] Karolchik D, Hinrichs AS, Furey TS, Roskin KM, Sugnet CW, Haussler D (2004). The UCSC table browser data retrieval tool. Nucleic Acids Res.

[42] Visel A, Minovitsky S, Dubchak I, Pennacchio LA (2007). VISTA enhancer browser - a database of tissue-specific human enhancers. Nucleic Acids Res.

[43] Curtin RR, Cline JR, Slagle NP, March WB, Ram P, Mehta NA, et al. MLPACK: a scalable C++ machine learning library. J Mach Learn Res. 2013;14:801–805. Available from: http://dl.acm.org/citation.cfm?id=2567709.2502606.

[44] Machanick P, Bailey TL (2011). MEME-ChIP: motif analysis of large DNA datasets. Bioinformatics.

[45] Marteijn JA, Lans H, Vermeulen W, Hoeijmakers JHJ. Understanding nucleotide excision repair and its roles in cancer and ageing. Nat Rev Mol Cell Biol. 2014;15:465–481. Available from: http://www.ncbi.nlm.nih.gov/pubmed/24954209. 10.1038/nrm3822 24954209

[46] Bogdanović O, Fernández-Miñán A, Tena JJ, de la Calle-Mustienes E, Gómez-Skarmeta JL (2013). The developmental epigenomics toolbox: ChIP-seq and MethylCap-seq profiling of early zebrafish embryos. Methods.

[47] Negre N, Brown CD, Ma L, Bristow CA, Miller SW, Wagner U (2011). A cis-regulatory map of the drosophila genome. Nature.

[48] Anders L, Guenther MG, Qi J, Fan ZP, Marineau JJ, Rahl PB (2014). Genome-wide localization of small molecules. Nat Biotechnol.

[49] Leinonen R, Sugawara H, Shumway M (2011). The sequence read archive. Nucleic Acids Res.

