# Supplementary material for: Software for rapid time dependent ChIP-sequencing analysis (TDCA)
Source: BMC Bioinformatics. 2017 Nov 25;18:521. doi: 10.1186/s12859-017-1936-x (PMC5702113; doi:10.1186/s12859-017-1936-x)
Supplement: Additional file 1: — SI for Software for Rapid Time Dependent ChIP-Sequencing Analysis (TDCA). (PDF 6149 kb) [file 12859_2017_1936_MOESM1_ESM.pdf]

Supplementary Figures and Legends

Software for Rapid Time Dependent ChIP-Sequencing Analysis (TDCA)

Mike Myschyshyn[1,*], Marco Farren-Dai[2], Tien-Jui Chuang[1] and David Vocadlo[1,2,*]

Department of [1]Molecular Biology and Biochemistry and [2]Chemistry, Simon Fraser University, 8888 University Drive, Burnaby, BC V5A 1S6, Canada.

*To whom correspondence should be addressed.

Contact: mmyschyshyn@gmail.com, dvocadlo@sfu.ca

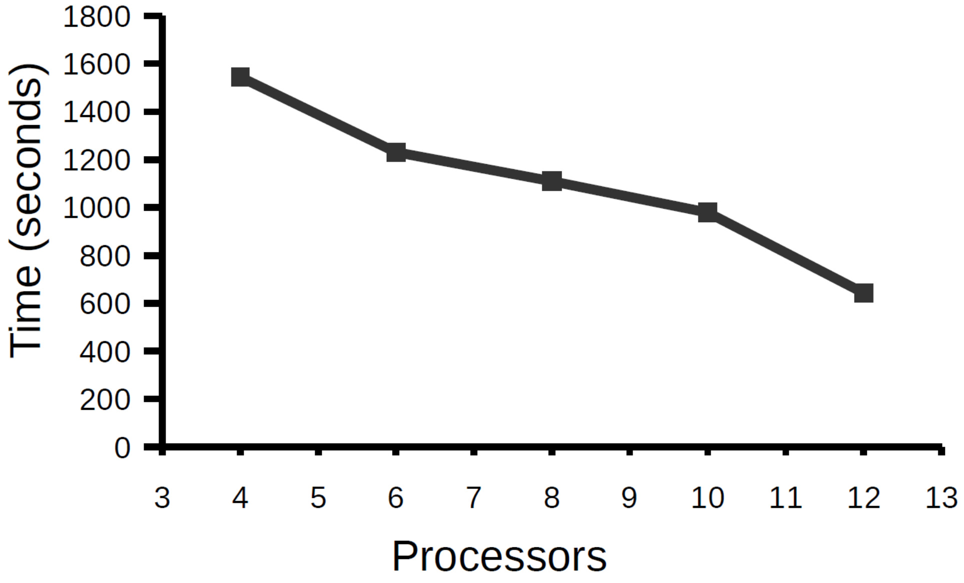

Supplementary Figure 1: TDCA is optimized to run on parallel processors. Processing times required for TC analysis of H3.3 bound loci on chromosome 10 using eleven time points as a function of variable numbers of processors used for computation. TDCA utilizes openmp to parallelize various algorithms in the program which requires an appropriate C++ compiler.

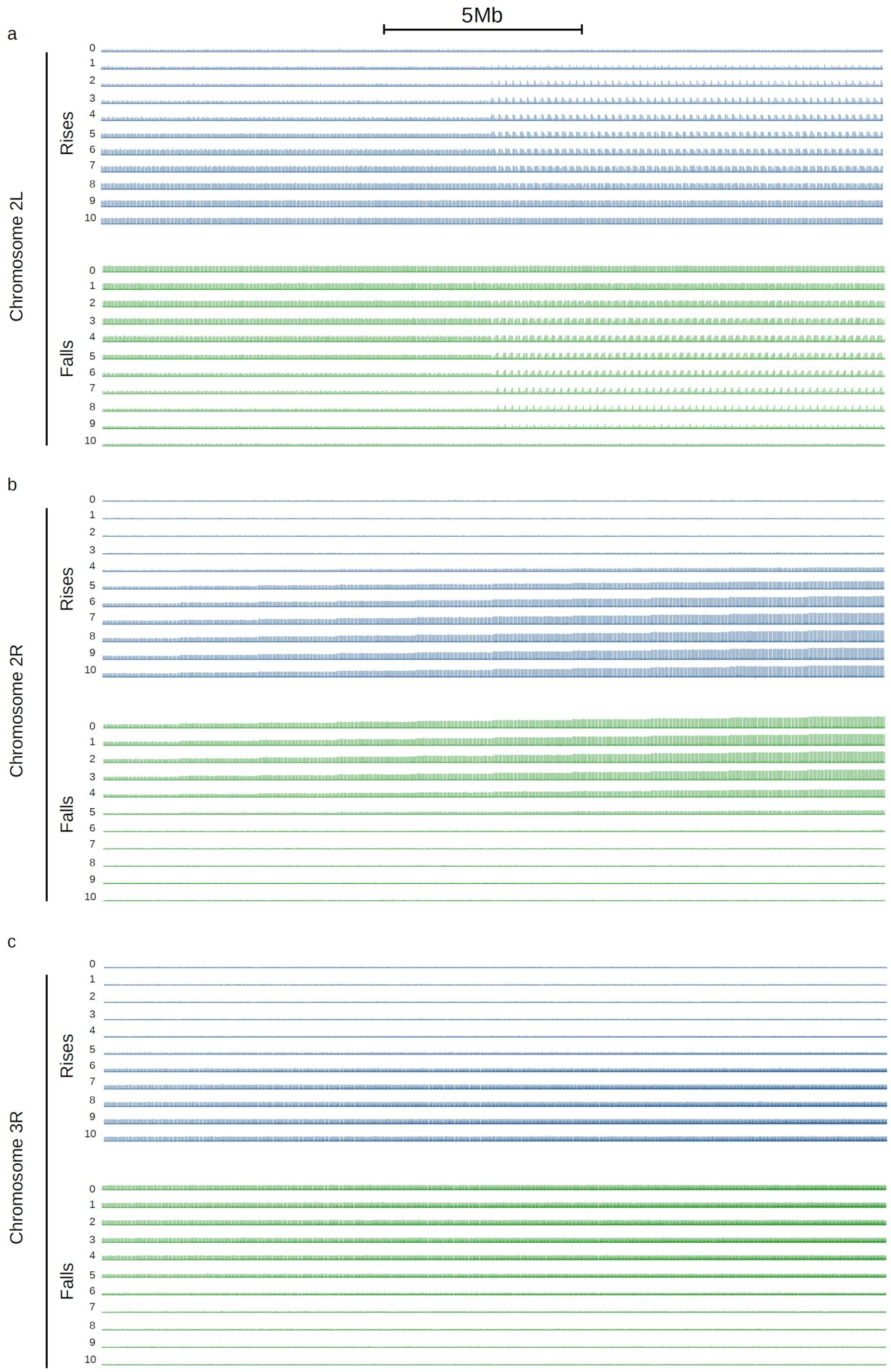

Supplementary Figure 2: UCSC snapshots of simulated data. Rises (blue) and falls (green) simulated data for chromosomes 2L (a), 2R (b), and 3R (c). Time of each ChIP-seq experiment is written to the left of each track (relative units). A total of 11 time points were generate: 0, 1, 2, 3, 4, 5, 6, 7, 8, 9, and 10. Scale bar are written above.

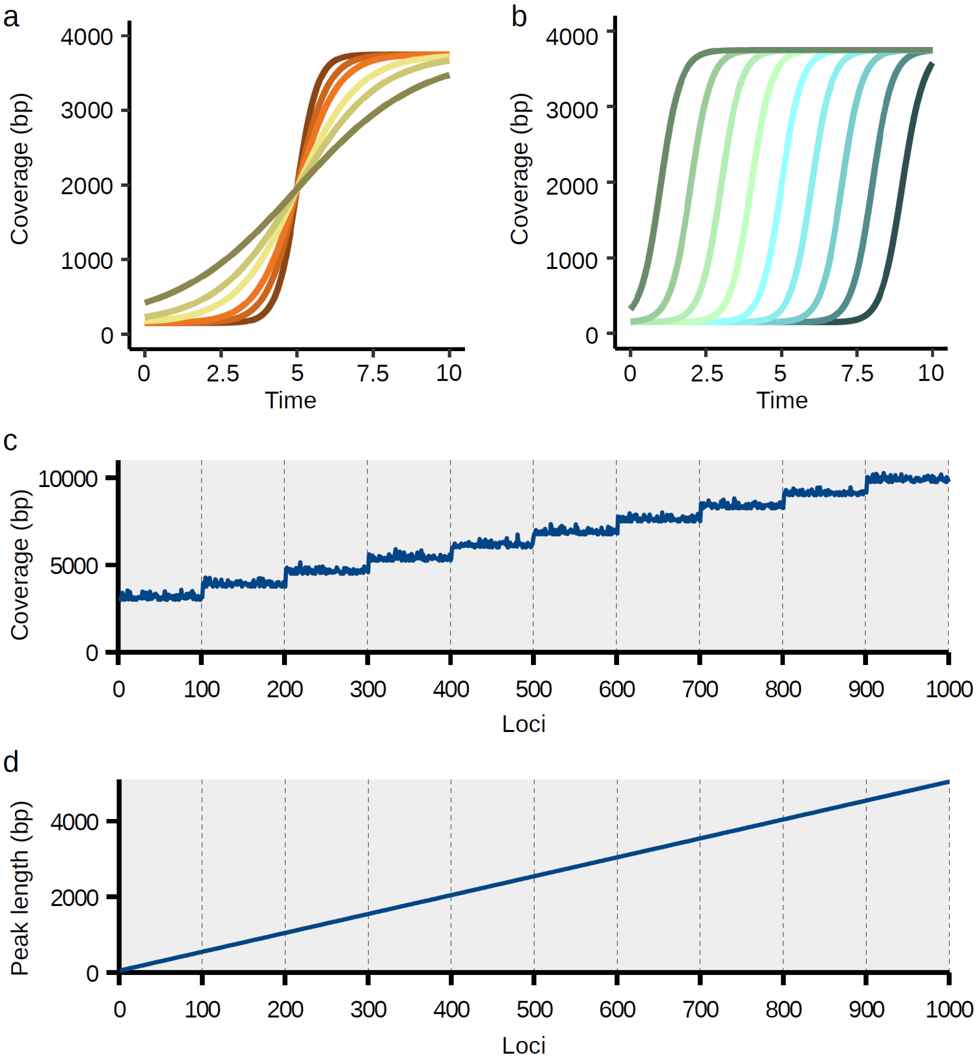

Supplementary Figure 3: Summary of simulated rise data. (a) Chromosome 2L contained 1000 loci with the first 500 loci containing variable incorporation rate index, resulting in data that behave as a sigmoid with steep and mellow slopes at the inflection point. Incorporation rate indices were set to: -0.5, -0.75, -1.0, -1.5, -2.0, and -3.0. (b) The second 500 loci of chromosome 2L contained loci with variable inflection points. Inflection points were set to: 1, 2, 3, 4, 5, 6, 7, 8, and 9 (relative time units). (c) Chromosome 2R contained 1000 loci of variable upper asymptote, shown here across loci. (d) Chromosome 3R contained 1000 loci of variable peak length, shown here across loci. Simulated fall data behaves similarly.

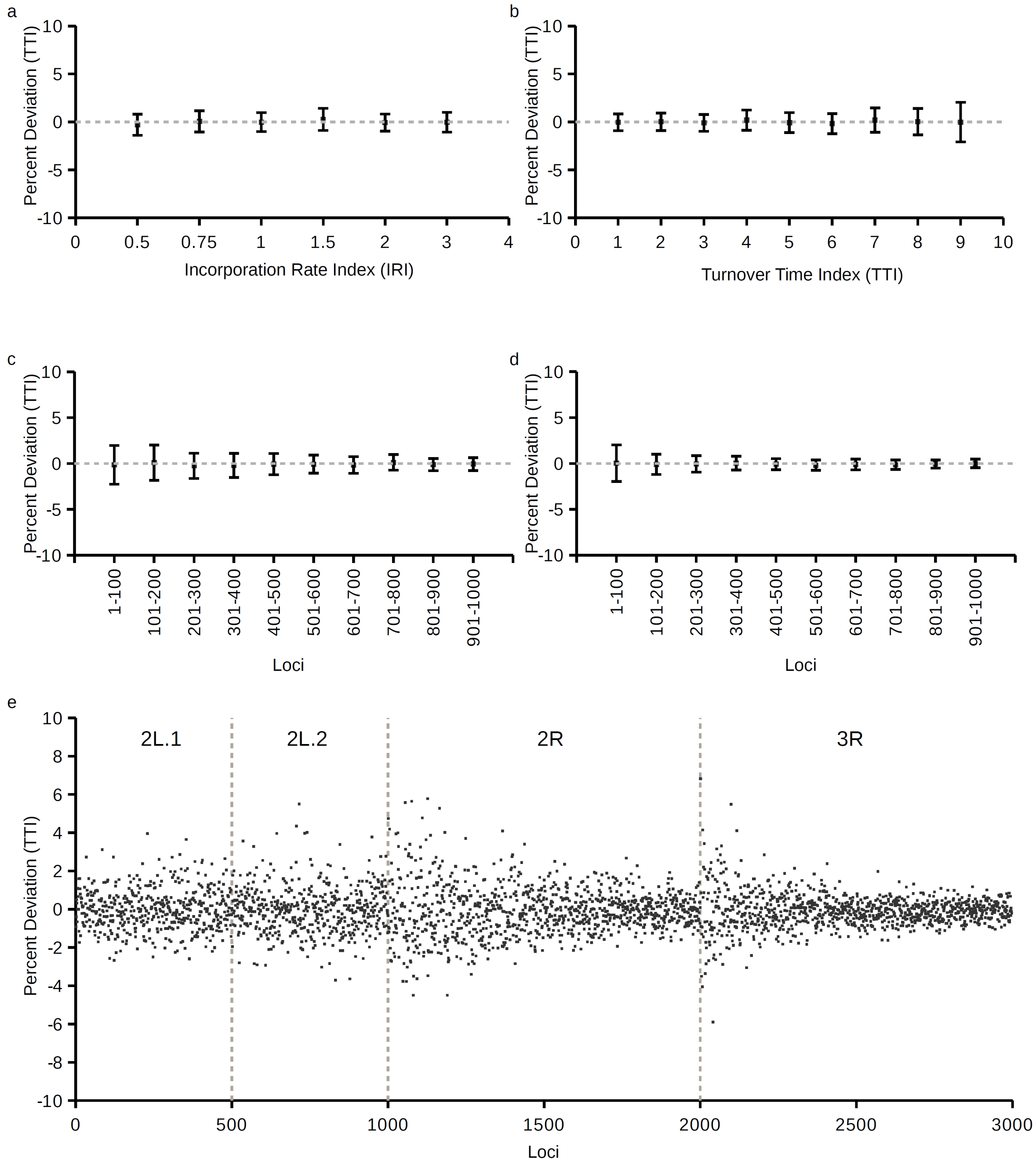

Supplementary Figure 4: Simulated rise data noise analysis. Noise was measured as a ratio of the sum of coverage across each time point for simulated data and the sum of expected coverage values across each time point, as a percent of expected coverage values. Average and standard deviation is shown for chromosome 2L.1 (a), chromosome 2L.2 (b), chromosome 2R (c), and chromosome 3R (d). Noise for each loci is shown in (e).

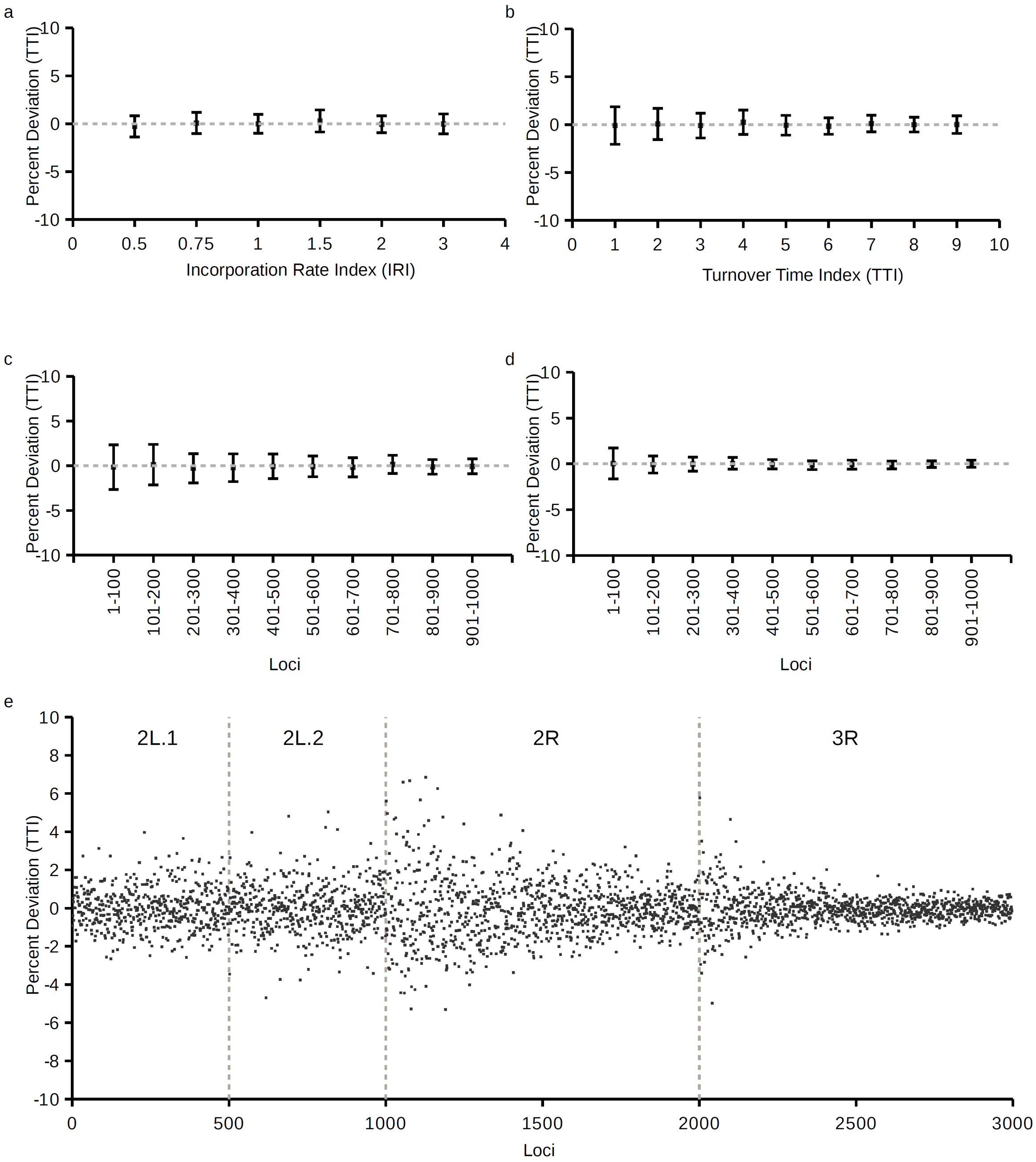

Supplementary Figure 5: Simulated fall data noise analysis. Same as in Supplementary Figure 4 except for simulated fall data.

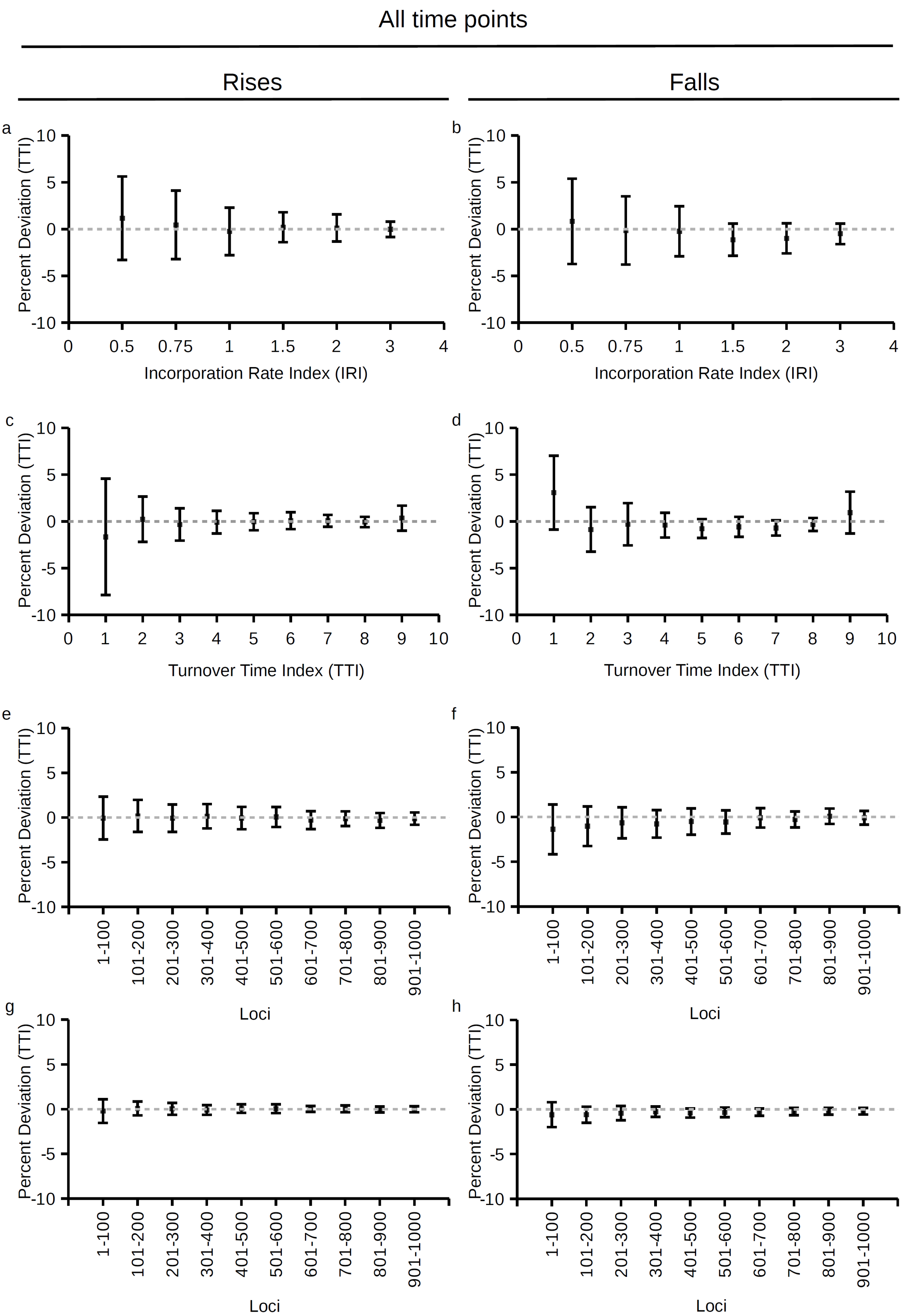

Supplementary Figure 6: Variance analysis of all time points. Percent deviation of TDCA modeled TTI to true TTI for rises (left) and falls (right). Data separated for chromosome 2L.1 (a-b), chromosome 2L.2 (c-d), chromosome 2R (e-f), and chromosome 3R (g-h).

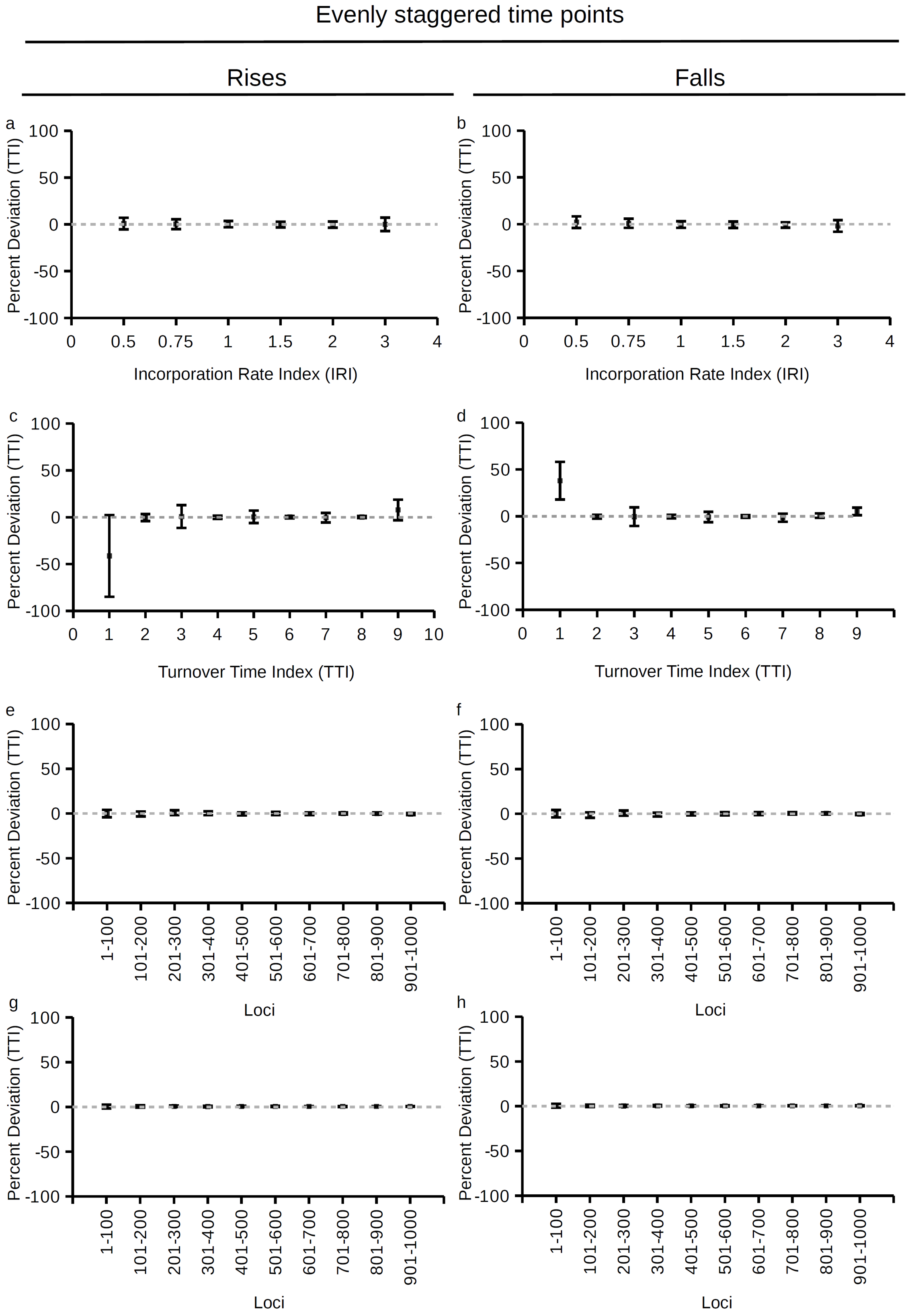

Supplementary Figure 7: Variance analysis as in Supplementary Figure 6 except for evenly staggered time points.

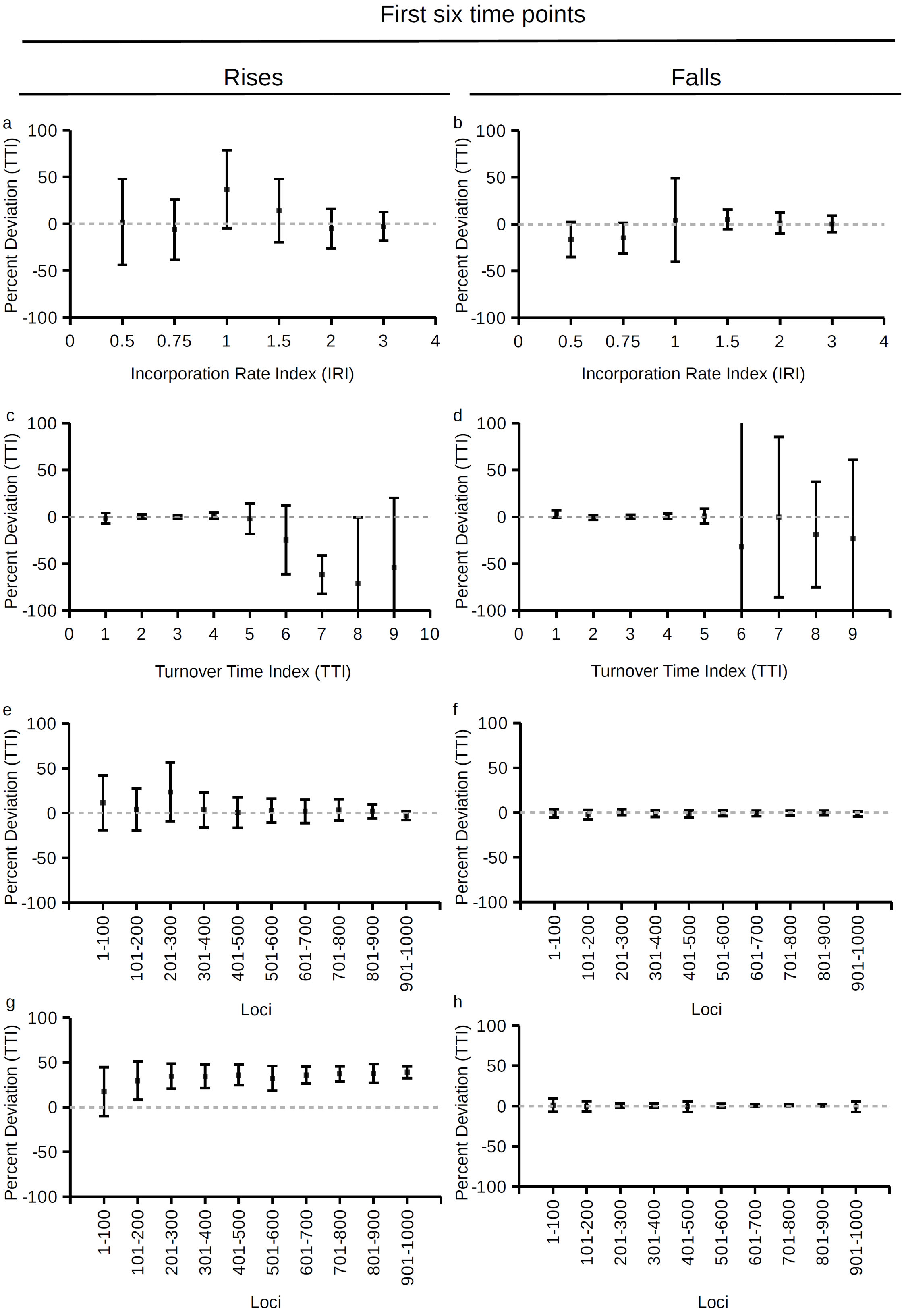

Supplementary Figure 8: Variance analysis as in Supplementary Figure 6 except for first six time points. Percent deviation for data point with inflection points 8 and 9 in plot (c) are 8: -71.3±70.6 and -54.1±74.3, respectively. Percent deviation for data point with inflection points 6 and 9 in plot (d) are -32.1±333.8 and -23.4±84.3, respectively.

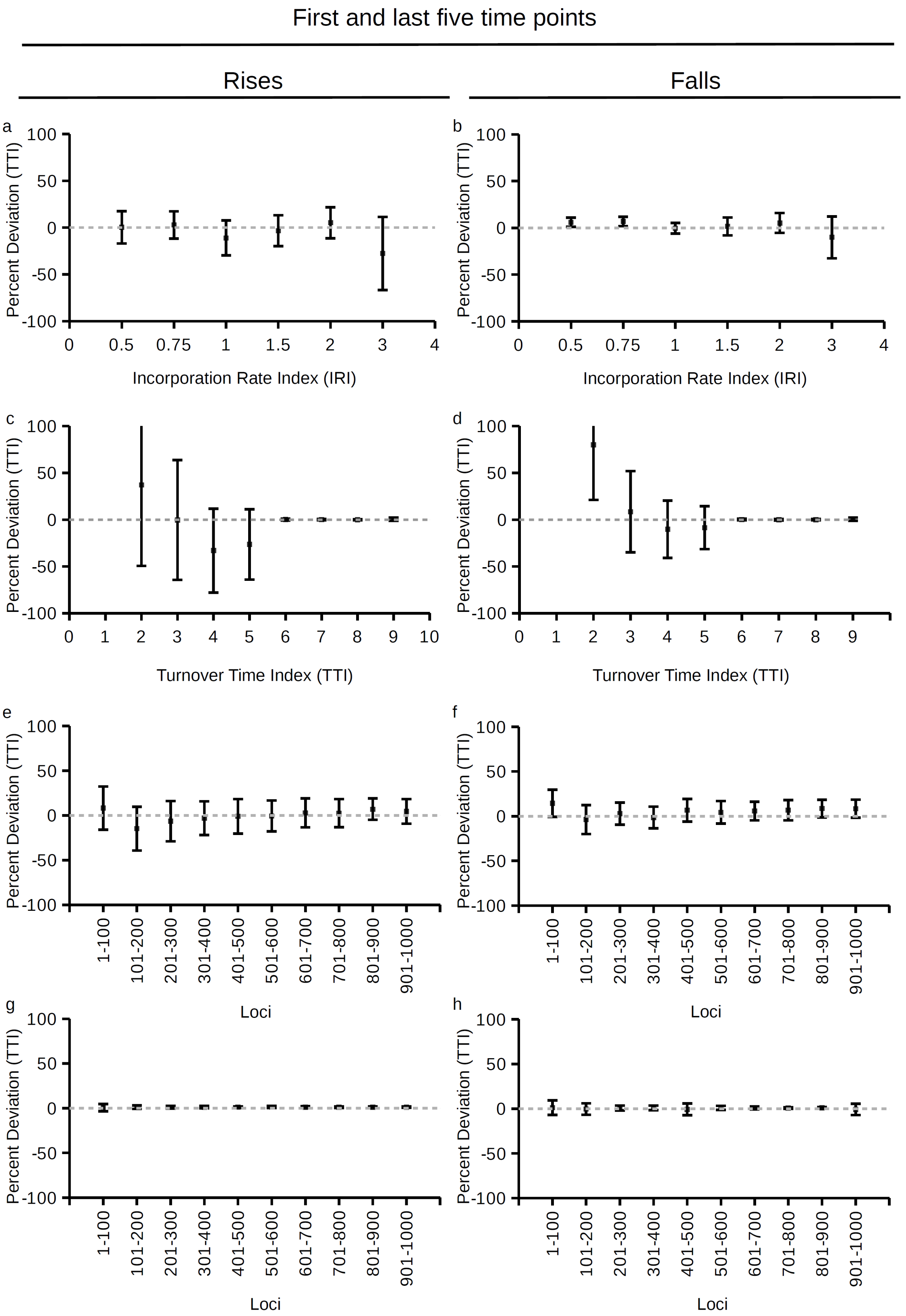

Supplementary Figure 9: Variance analysis as in Supplementary Figure 6 except for first and last five time points. Percent deviation for data point with inflection points 1 and 2 in plot (c) are 159.1±168.4 and 37.2+±86.7, respectively. Percent deviation for data point with inflection points 1 and 2 in plot (d) are 264.2±122.0 and 80.0±59.0, respectively.

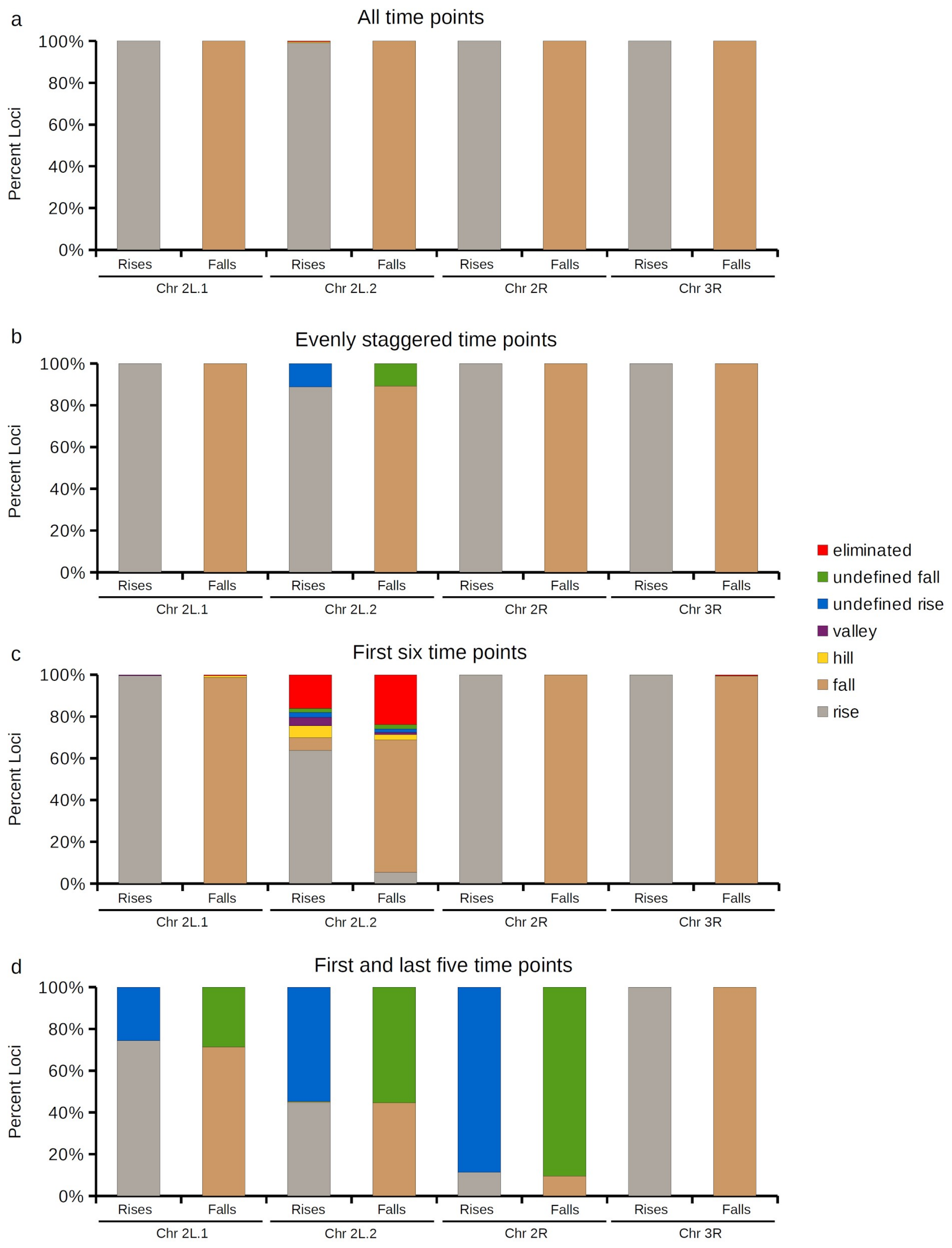

Supplementary Figure 10: Loci category identification in simulated data. Stacked bar charts of rises and falls separated by chromosomes. Y-axis indicates percent loci. Proportion of loci category are shown for all (a), staggered (b), first six (c), and first and last five (d) time points.

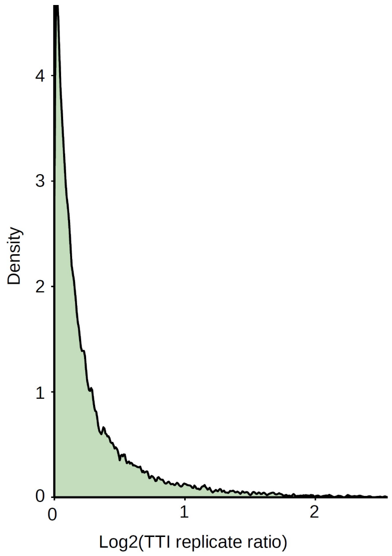

Supplementary Figure 11: Replicate analysis of H3.3 TC data. Density plot of log2 ratio of replicate 1 TTI/ replicate 2 TTI. 73.4% of loci are within 20% TTI ratio of each other and 94.4% of loci are within 50% TTI ratio of each other.

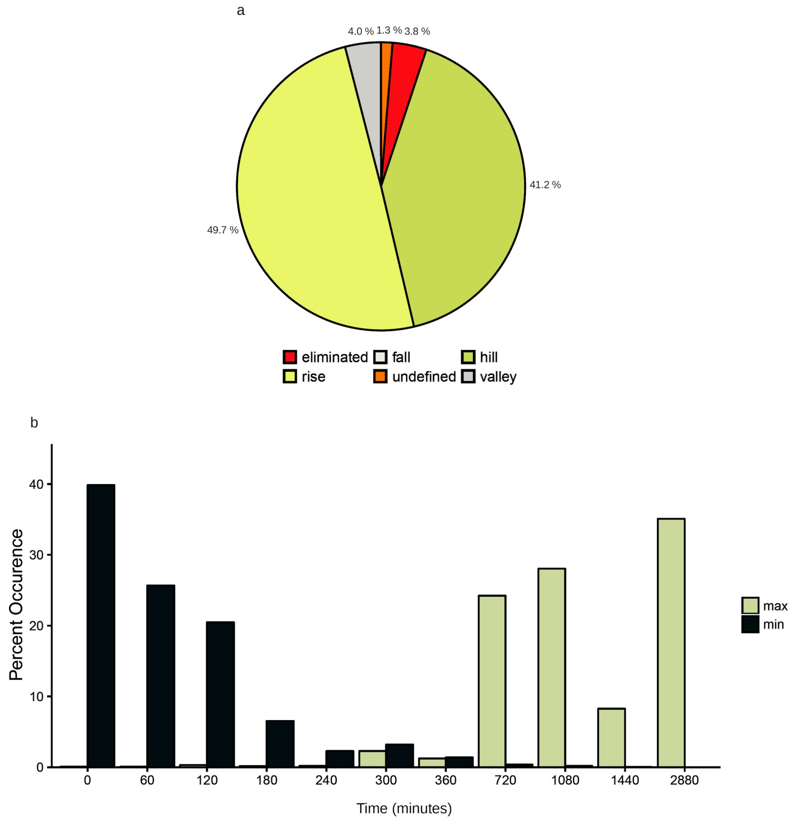

Supplementary Figure 12: Quality analysis of H3.3 TC data. (a) Pie chart of loci separated by different model categories. (b) Bar chart showing percent occurrence of absolute minimum (min) and absolute maximum (max) coverage values of all loci.

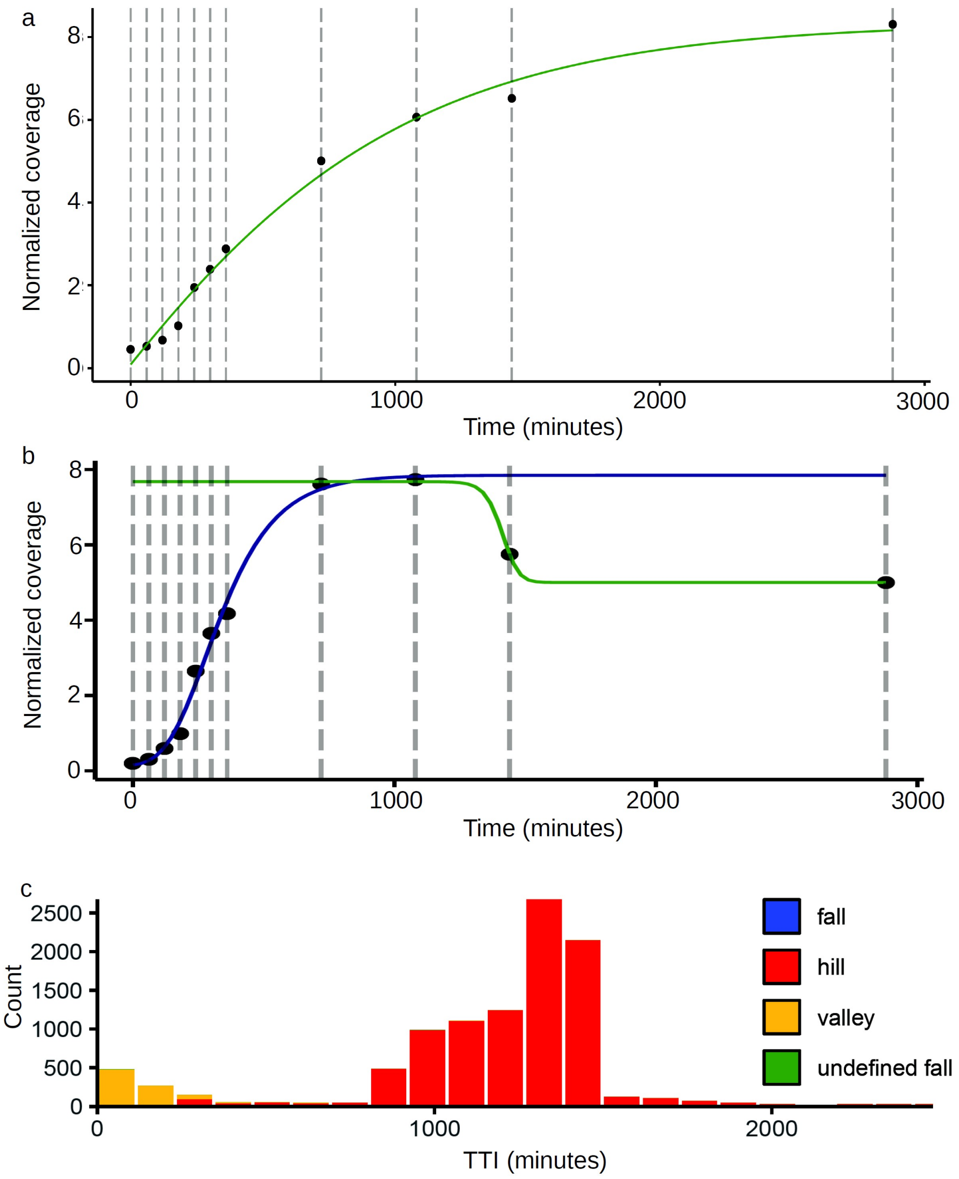

Supplementary Figure 13: Behavior and genomic distribution of H3.3 TC data. (a) Average profile of loci that model as rises. Time in minutes is shown on the x-axis. Coverage at each time point are shown as black dots. Normalized coverage is characterized as the ratio of the delta coverage averaged across all loci that behave as rises. (b) Average profile of loci that model as hills. The curve that models the incline of hills is shown in blue and the curve that models the decline in green. Time in minutes is shown on the x-axis. Coverage at each time point are shown as black dots. Normalized coverage is characterized as the ratio of the delta coverage averaged across all loci that behave as hills. (c) Distribution of loci that display signal decrease grouped as different categories. TTI is shown on the x-axis and loci count on the y-axis.

Sgk1 (chr10:21712028-21719708)

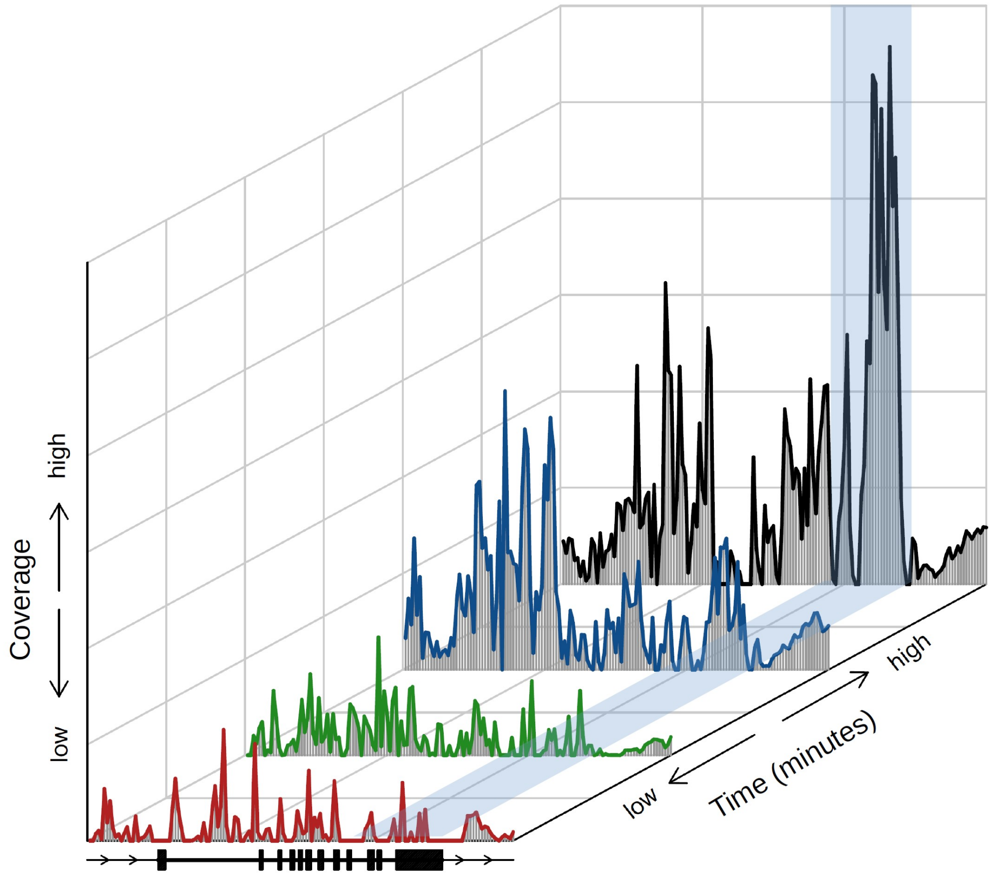

Supplementary Figure 14: 3D plot of sequencing coverage for Sgk1 (chr10:21712028-21719708). Black boxes show exons, dark lines introns, and lines with arrows are 1000bp upstream and downstream regions. Highlighted region shows the position of two loci with TTI values of 1868.4 and 1732.5 minutes.

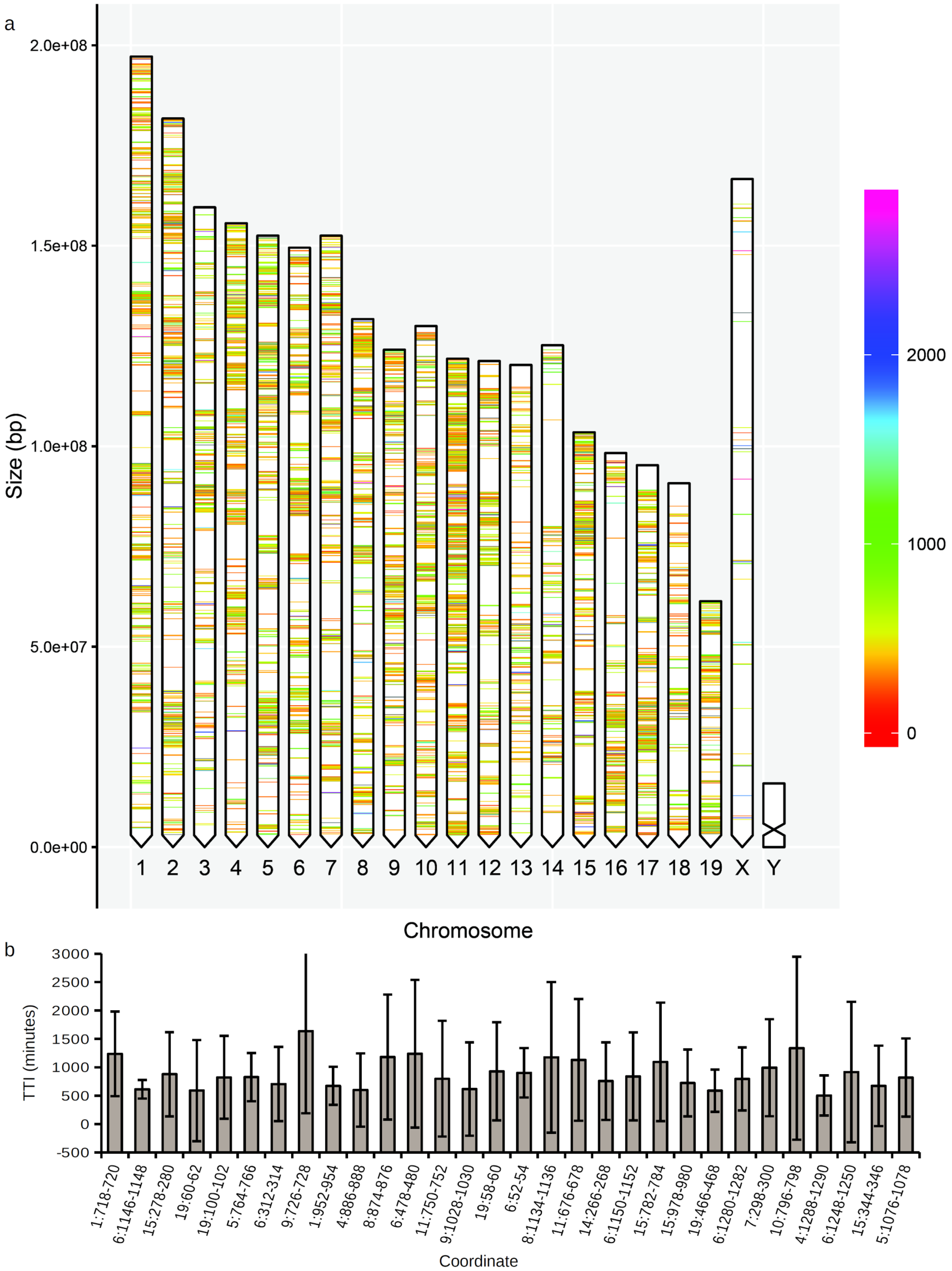

Supplementary Figure 15: Behavior and genomic distribution of H3.3 TC data. (a) Ideogram heat map. Bands indicate where H3.3 is bound and the color scale indicates the TTI value. (b) Average and standard deviation of TTI (minutes) of 200,000 bp clusters that contain 30 or more loci (clusters). Coordinates are written as chromosome: start (base pairs e-5) - end (base pairs e-5).

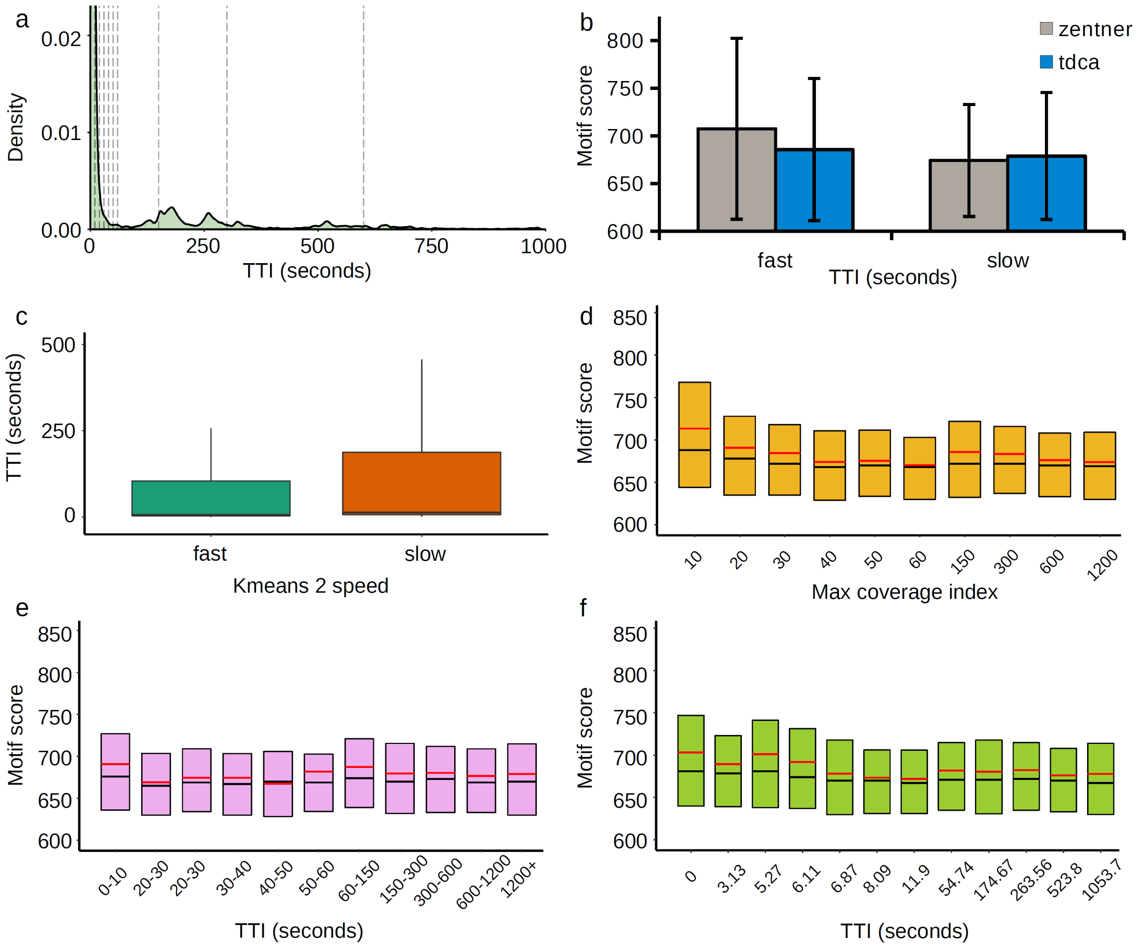

Supplementary Figure 16: Chec-seq analysis of Abf1 hills and rises in yeast. (a) Distribution of TTI values (seconds) of loci. (b) Average motif scores of loci that were originally categorized as fast and slow by k-means = 2 clustering (zentner) [1] and loci clustered by k-means = 2 by TTI (tdca). (c) Distribution of TTI values in the originally categorized fast and slow loci. (d) Distribution of motif scores of loci categorized by time point (seconds – x-axis) at which the absolute maximum depth occurs. Black midline indicates median and red midline indicates average. Quartiles 2 and 3 are lower and upper fractions of the box divided by the median. (e) Distribution of motif scores of loci binned by the time point used in the TC experiment. Black midline indicates median and red midline indicates average. Quartiles 2 and 3 are lower and upper fractions of the box divided by the median. (f) Distribution of motif scores of loci ordered from fastest to slowest TTI and binned into groups of 1000. TTI in seconds is shown on the x-axis. Black midline indicates median and red midline indicates average. Quartiles 2 and 3 are lower and upper fractions of the box divided by the median.

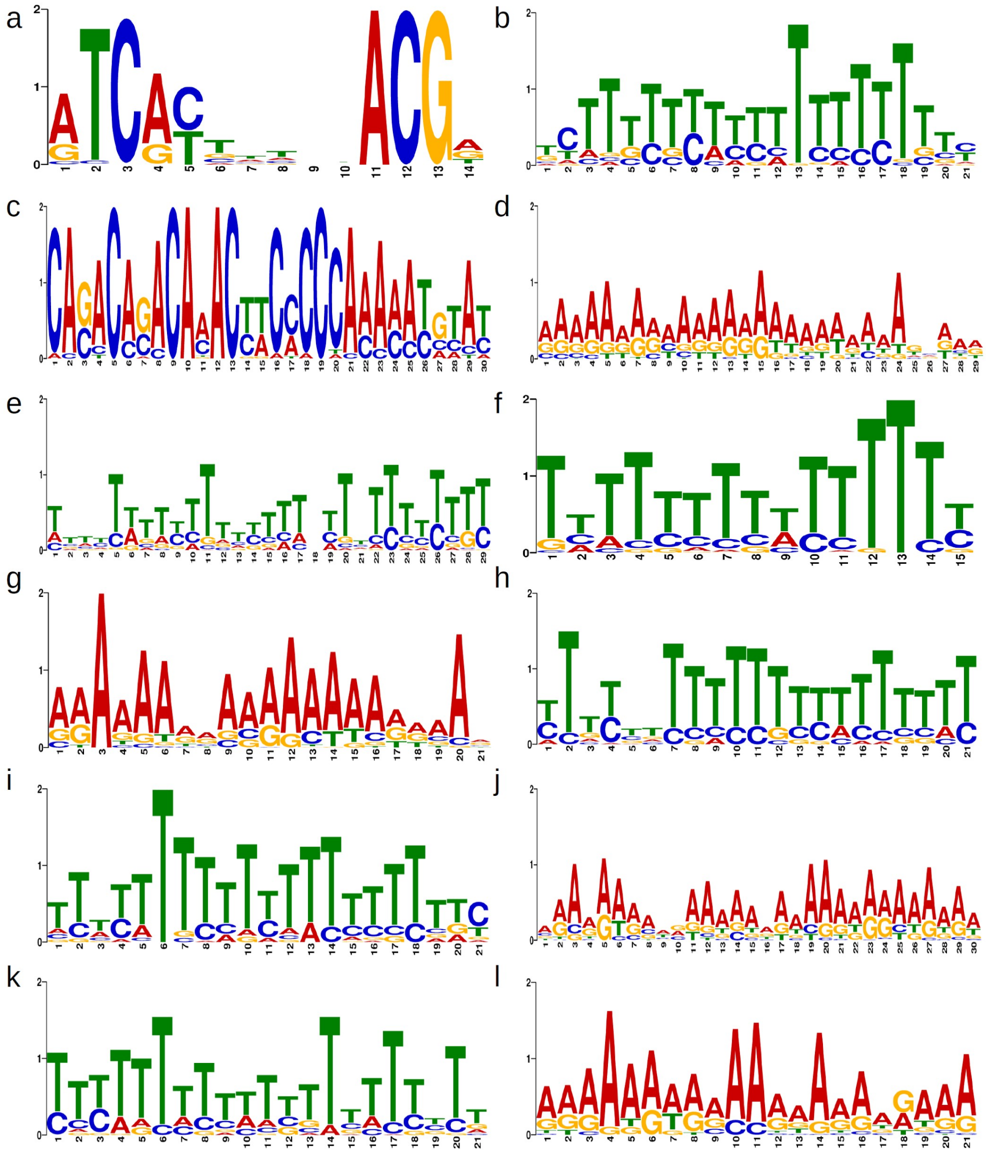

Supplementary Figure 17: Abf1 Chec-Seq motifs. Top scoring motifs (most significant) of loci ordered from fastest to slowest TTI and binned into groups of 1000 (a-l).

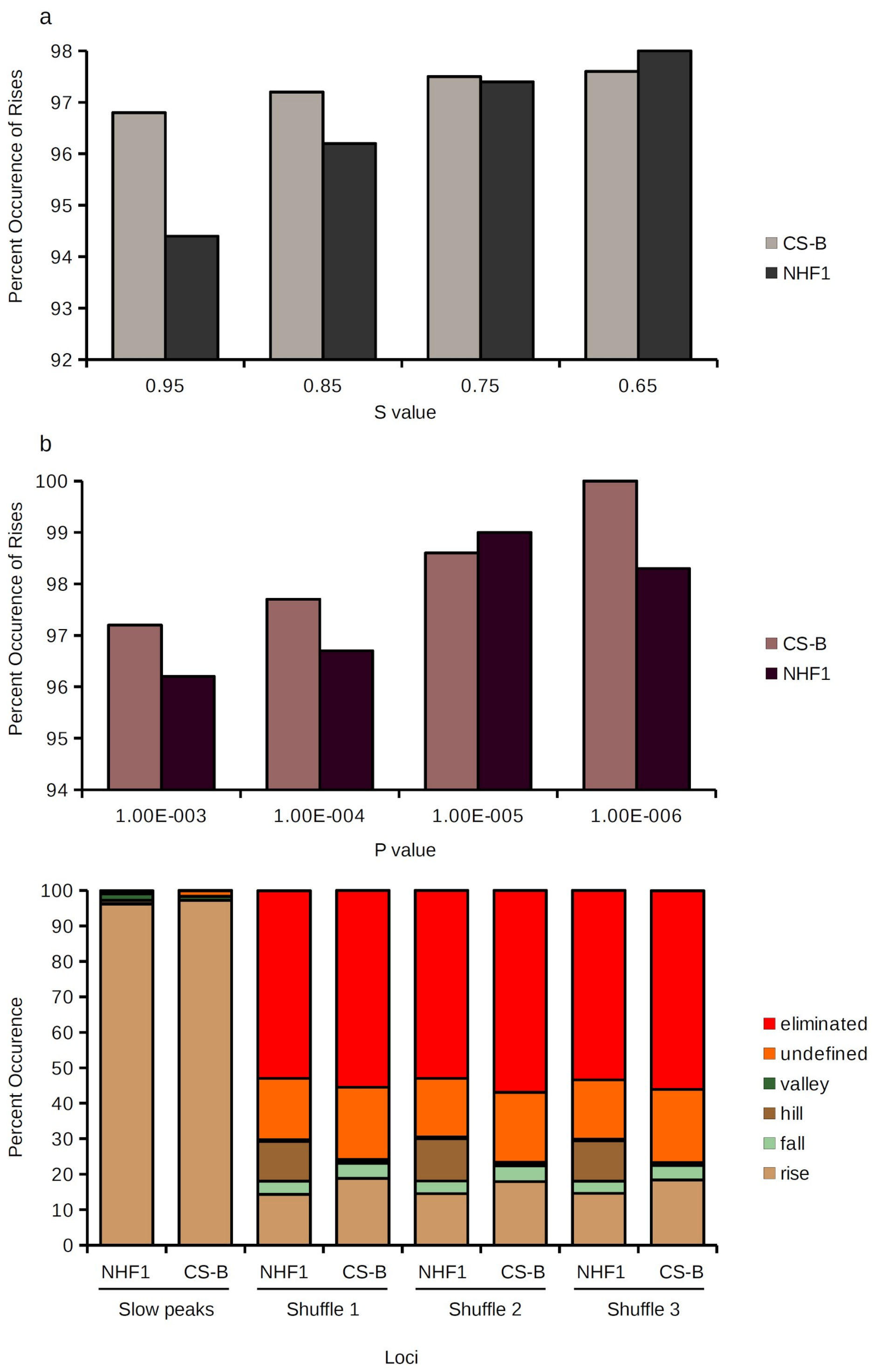

Supplementary Figure 18: Analysis of slow [6-4]PP XR-Seq loci. (a) Percent occurrence of rises as the TDCA plateau range threshold (s-value) is altered. (b) Percent occurrence of rises as the macs peak calling p-value is altered. (c) Category of loci modeled using constant normalization values (see -dm flag in manual) in slow peaks and loci that were generated by shuffling the positions of the slow peaks in three random operations (shuffle 1 to 3).

References

1. Zentner GE, Kasinathan S, Xin B, Rohs R, Henikoff S. ChEC-seq kinetics discriminates transcription factor binding sites by DNA sequence and shape in vivo. Nat. Commun. Nature Publishing Group; 2015;6:8733.
